# Supramolecular Hydrogels from a Tripeptide and Carbon Nano-Onions for Biological Applications

**DOI:** 10.3390/nano13010172

**Published:** 2022-12-30

**Authors:** Davide Marin, Michał Bartkowski, Slavko Kralj, Beatrice Rosetti, Paola D’Andrea, Simone Adorinni, Silvia Marchesan, Silvia Giordani

**Affiliations:** 1 Chemical and Pharmaceutical Sciences Department, University of Trieste, 34127 Trieste, Italy; 2 School of Chemical Sciences, Faculty of Science & Health, Dublin City University, D09 E432 Dublin, Ireland; 3 Department for Materials Synthesis, Jožef Stefan Institute, 1000 Ljubljana, Slovenia; 4 Department of Pharmaceutical Technology, Faculty of Pharmacy, University of Ljubljana, 1000 Ljubljana, Slovenia; 5 Department of Life Sciences, University of Trieste, 34127 Trieste, Italy

**Keywords:** carbon nano-onions, carbon nanomaterials, peptides, self-assembly, supramolecular chemistry, gels, biomaterials, hydrogelation, D-amino acids, nanocomposites

## Abstract

Nanocomposite hydrogels have attracted researchers’ attention in recent years to achieve superior performances in a variety of materials applications. In this work, we describe the outcome of three different strategies to combine a self-assembling tripeptide and carbon nano-onions (CNOs), through covalent and non-covalent approaches, into supramolecular and nanostructured hydrogels. Importantly, the tripeptide coated the nano-onions and extended their aqueous dispersions’ stability by several hours. Furthermore, CNOs could be loaded in the tripeptide hydrogels at the highest level ever reported for nanocarbons, indicating high compatibility between the components. The materials were formed in phosphate-buffered solutions, thus paving the way for biological applications, and were characterized by several spectroscopic, microscopic, thermogravimetric, and rheological techniques. In vitro experiments demonstrated excellent cytocompatibility.

## 1. Introduction

Nanocomposites are multiphase materials that incorporate structures that, by definition, have dimensions in the nanoscale. Recently, we have witnessed the rise of nanocomposites, thanks to their great versatility for various applications [1]. The inclusion of different nanosized components offers a convenient approach to modulate the material properties, whilst taking advantage of nanotechnology for a superior performance, relative to conventional materials. In particular, carbon nanomaterials (CNMs) have attracted academic and industrial interest for their unique physico-chemical properties [2] and for the possibility of their fine-tuning through chemical derivatization [3,4]. Furthermore, interest in CNMs has been compounded by the wide breadth of potential uses, which encompass the diverse fields of energy [5,6,7], catalysis [8,9,10], environmental remediation [11,12,13,14], automotive and aerospace transport [15,16,17], tissue engineering [18,19,20,21,22], targeted drug delivery [23,24,25], theranostics [26,27,28], sensing [29,30,31], imaging [32,33,34], and flexible electronics [35,36,37]. A prominent industrial application of carbon nanostructures is their use in the automotive industry—when used as a nanoadditive in tyre manufacture, the resulting nanocomposite exhibits significantly increased durability [38].

Soft matter, such as hydrogels, can benefit from the inclusion of CNMs to gain new mechanical, thermal, and electronic properties [39,40,41,42,43]. Amongst the many hydrogelators, short peptides are popular building blocks for their biodegradability and environmental compatibility, as well as for offering the possibility to encode biological messages [44] and to program their enzymatic conversion into active or inactive species by design [45]. These scaffolds are ideal biomaterials to mimic the extracellular matrix and promote cell proliferation and growth for tissue regeneration [44,45]. Shorter peptide-based hydrogelators are easier and cheaper to produce, with di- and tri-peptides being ideal candidates for low-cost liquid-phase synthesis at small and large scales [46]. In particular, sequences that exploit the diphenylalanine motif are very attractive for their strong self-assembly propensity [47,48,49], and for the possibility to establish hydrophobic interactions with CNMs into functional composites [50,51,52].

Despite the increasing interest in the emerging area of nanocomposites incorporating both CNMs and self-assembling short peptides [53], to the best of our knowledge, no such studies thus far have focused on carbon nano-onions (CNOs). CNMs are generally hydrophobic in nature, and their poor dispersibility in aqueous environments to attain homogeneous nanocomposite hydrogels is a typical problem [53]. CNOs are multi-layered nanostructures comprised of concentric fullerenes—they were first prepared by Ugarte in 1992 [54]. The chemistry of CNOs is well-established; there are many possible routes for CNO-surface functionalization, which allows for fine-tuning the nanostructures’ properties [55]. Furthermore, their excellent cytocompatibility makes them attractive components for biological uses [56,57], such as bioimaging [58], drug delivery [59], and vehicles for biomolecules [60].

In this work, we explored three functionalization routes to combine CNOs with a self-assembling short peptide into hydrogels. One key aspect to bear in mind is the dispersibility of the CNM in the solvent of choice, which is required to ensure the final nanocomposite’s best performance. With the choice of solvent in consideration, the CNO-peptide hydrogels were prepared in phosphate buffer. These solutions are physiologically compatible and could pave the way for future applications of CNO-peptide hydrogels as nanocomposite biomaterials. Indeed, in previous studies, CNOs displayed an excellent in vitro [59,61] and in vivo [62,63] biocompatibility profile and have been utilized as components for bioimaging and sensing [58,64,65]. In this work, we identify the most promising route to prepare nanocomposite hydrogels from a self-assembling tripeptide and CNOs, we demonstrate the colloidal stability of the nanoparticles thanks to their surface coating by the peptide, as well as the hydrogels’ stability in physiological conditions and excellent cytocompatibility in vitro.

## 2. Materials and Methods 

### 2.1. Materials and General Methods

All solvents and reagents were acquired from Merck (Milan, Italy) and used as received unless otherwise specified. High-purity Milli-Q water with a minimum resistivity of 18.2 MΩ·cm @ 25 °C was produced from a Milli-Q Academic System (Millipore RiOs/Origin purification system, St. Louis, MS, USA) and employed to prepare all solutions and buffers. The microwave (MW)-assisted synthesis was carried out in the Microwave reactor Discover SP–CEM Corporation (Bergamo, Italy). The sonicator used was the Branson Ultrasonic 3800 cleaning bath (Milan, Italy).

### 2.2. Thermogravimetric Analysis (TGA)

Thermogravimetric analyses (TGA) were carried out on a TGA5500 (TA Instruments, Milan, Italy) under a nitrogen and an air atmosphere. For each sample, 1 mg of material was analyzed by equilibrating at 100 °C for 20 min and then applying a heating ramp of 10 °C min^–1^ up to 800 °C.

### 2.3. Infrared Spectroscopy

Attenuated total reflectance Fourier-transform infrared spectroscopy (ATR-FTIR) spectra were acquired at 4 cm^−1^ resolution, 240 scans, with an ATR-FTIR Affinity-1S (Shimadzu, Milan, Italy). Every sample was analyzed at least three times.

### 2.4. Raman Analysis

Samples were prepared on microscopy glass slides and dried overnight. Raman spectra were acquired on five different spots per sample with an inVia Renishaw microspectrometer (50, Turin, Italy) equipped with laser at 532 nm (25 mW), with 1 accumulation, 1 cm^−1^ resolution. Raman mapping was carried out based on the intensity ratio of the signals at 1351 and 1002 cm^−1^.

### 2.5. Transmission Electron Microscopy (TEM)

Transmission electron microscopy (TEM) was carried out on a JEM 2100 (Jeol, Tokyo, Japan) at 100 kV. Samples were transferred onto TEM carbon grids previously exposed to UV-Ozone Procleaner Plus for 6 min and dried in vacuo. The grid samples were stained with potassium phosphotungstate (2% pH 7.2). Electron micrographs were analyzed with FIJI free software (ImageJ 2).

### 2.6. Oscillatory Rheology

Rheological analyses were performed on a Malvern Kinexus Ultra Plus Rheometer (Alfatest, Milan, Italy) using a 20 mm stainless-steel, parallel-plate geometry with a 1 mm gap at a fixed temperature of 25 °C using a Peltier temperature controller (Alfatest, Milan, Italy). The hydrogels were formed directly on the rheometer plate by mixing 200 μL of the CNOs dispersion in the peptide solution in the alkaline buffer and the same amount of the acidic buffer. Time sweeps were performed at 1 Pa and 1 Hz for 1 h. Frequency sweeps were recorded at 2 Pa from 0.1 to 10 Hz. Stress sweeps were performed from 1 Pa up to gel failure at 1 Hz.

### 2.7. HPLC and LC-MS

All reverse-phase HPLC purifications were carried out on the 1260 Agilent Infinity system equipped with a preparative gradient pump (G1311B), semipreparative C-18 column (Kinetex, 5 μm, 100 Å, 250 × 10 mm, Phenomenex, Milan, Italy) and the photodiode array detector (G1315C) set at 214 and 254 nm. All LC-MS analyses were carried out on the 6120 Agilent Infinity system that consists of the 1260 described above connected to the ESI-MS detector, single quadrupole.

### 2.8. Nuclear Magnetic Resonance (NMR)

NMR spectra were acquired on a Varian 400 MHz (Milan, Italy) at 101 MHz for ^13^C and 400 MHz for ^1^H nuclei. Samples were dissolved in deuterated solvents as indicated in the Supplementary Materials File and chemical shifts reported in ppm relative to tetramethylsilane (TMS) as reference.

### 2.9. Pristine CNOs (p-CNOs) Preparation

Detonation nanodiamond powder (uDiamond^®^ Molto from Carbodeon Ltd., Vantaa, Finland; 5 nm average particle size) was thermally annealed at 1650 °C under a positive pressure of helium to produce small p-CNOs (6–7 nm in size; 6–8 shells). The procedure was carried out as previously reported [66,67].

### 2.10. Oxidized CNOs (Oxi-CNOs) Preparation

p-CNOs were dispersed in 3 M nitric acid by sonication. The dispersion was then stirred under reflux at 110 °C for 48 h. After cooling to room temperature (RT), excess nitric acid was removed through centrifugation, removal of the supernatant, and redispersion of the material in deionized water. The oxi-CNOs were then filtered off on a Millipore membrane (GNWP, 0.2 μm) and sequentially washed with water, methanol, and acetone. After drying overnight at RT, the oxi-CNOs were recovered as black powder [61].

### 2.11. PEGylated CNOs (Amino-PEG-CNOs) Synthesis

The amino-terminated PEGylated CNOs (amino-PEG-CNOs) were prepared as previously described [61]. PEGylation of oxi-CNOs with 4,7,10-trioxa-1,13-tridecanediamine (diamino-PEG) was carried out through an *N*-hydroxysuccinimide/1-(3-dimethylaminopropyl)-3-ethylcarbodiimide hydrochloride (NHS/EDC·HCl) mediated amidation coupling in dry *N*,*N*-dimethylformamide (DMF), with 4-dimethylaminopyridine (DMAP) added as a catalyst. The coupling reaction was carried out with stirring under reflux at 140 °C for 3 days. After cooling to RT, the amino-PEG-CNOs were filtered off on a Millipore membrane (GNWP, 0.2 μm) and sequentially washed with DMF, water, methanol, and acetone. After drying overnight at RT, the amino-PEG-CNOs were recovered as black powder.

### 2.12. L-Leu-D-Phe-D-Phe (Lff) Synthesis

The tripeptide L-Leu-D-Phe-D-Phe (Lff) was synthesized by solid phase according to published procedures, using 2-chlorotrityl chloride resin [68]. Lff was purified by reversed-phase HPLC. Water and acetonitrile (MeCN), both with 0.05% of trifluoroacetic acid (TFA), were used as solvents. The reactions’ crudes were first dissolved into a mixture of MeCN/water 30:70 (with 0.05% of TFA in both solvents), and then the mixture was filtered with a 0.45 μm PTFE filter. The following HPLC method was used: t = 0–2 min, 30% MeCN; t = 16 min, 95% MeCN; t = 18 min, 95% MeCN (Rt = 8 min).

### 2.13. Fmoc-Lff Synthesis

The Lff Fmoc *N*-protection strategy to form Fmoc-Lff was carried out as per published procedures [68]. In contrast to Lff, Fmoc-Lff was not soluble in water or MeCN; consequently, it was purified by trituration instead of reverse-phase HPLC. The crude was washed using MeCN/water 30:70 (with 0.05% of TFA in both solvents) and filtered on a Millipore membrane (JHWP, 0.45 μm). The so obtained pale-orange solid was rewashed on the filter until a white solid was obtained.

### 2.14. PEGylated Lff (Amino-PEG-Lff) Synthesis

To prepare the amino-terminated PEGylated Lff, Fmoc-Lff was prepared as described above—however, prior to cleaving the product off the resin, leucine was deprotected and the Fmoc-8-amino-3,6-dioxaoctaoic acid (Sigma, Milan, Italy) PEG linker was added by treating it just like an amino acid, and then likewise it was Fmoc-deprotected and the product was cleaved off the resin through acidic hydrolysis. Amino-PEG-Lff was purified by dissolving it in MeCN/water 30:70 (with 0.05% of TFA in both solvents) and then the mixture was filtered with a 0.45 μm PTFE filter and injected in the HPLC using the system described above (1260 Infinity system, Agilent, Milan, Italy) and the following method: t = 0–2 min, 20% MeCN; t = 152 min, 50% MeCN; t = 153 min, 95% MeCN; t = 158 min 95% MeCN (Rt = 53 min).

### 2.15. Lff-PEG-CNOs (C-Terminus) Synthesis

Amino-PEG-CNOs (15 mg) were placed in a round-bottom flask and sonicated for 30 min in DMF (13 mL). Next, a solution of Oxyma Pure B (3 eq), *N*,*N*′-diisopropylcarbodiimide (DIC) (3 eq) and Fmoc-Lff (3 eq) in DMF (2 mL) was added, and the final mixture was kept under vigorous magnetic stirring for 24 h at RT. The crude was filtered on a Millipore membrane (JHWP, 0.45 μm) and washed with DMF three times, twice with DCM, water and methanol, and then with diethyl ether. The product was left to dry overnight on the filter. For the MW-assisted coupling, amino-PEG-CNOs (10 mg) were placed in a MW reactor vessel and dispersed in DMF (2 mL) by sonication (30 min). A solution of Oxyma Pure B (3 eq), DIC (3 eq), and Fmoc-Lff (3 eq) in 1 mL of DMF was added, and the final mixture was put in the MW reactor for 15 min at 80 °C, with a power of 150 W. After that time, the crude was filtered on a Millipore membrane (JHWP, 0.45 μm) and washed as indicated above. The deprotection was performed by stirring the coupling product for 30 min at RT in 20% piperidine in DMF (10 mL). The final product was filtered, washed, and dried as indicated above.

### 2.16. Lff-PEG-CNOs (N-Terminus) Synthesis

Oxi-CNOs (10 mg) were placed in a round-bottom flask and sonicated for 30 min in DMF (6 mL). A solution of Oxyma Pure B (5 eq) and DIC (3 eq) in DMF (2 mL) was added, and the resulting mixture was kept under vigorous magnetic stirring for 16 h at RT. Next, a solution of amino-PEG-Lff (5 eq) in DMF (2 mL) was added, and the final mixture was kept under vigorous magnetic stirring for 24 h at RT. The crude was filtered on a Millipore membrane (JHWP, 0.45 μm) and washed with DMF three times, DCM, water and methanol two times for each solvent, and the last time with diethyl ether, and the product was left to dry overnight at RT.

### 2.17. Self-Assembly into Nanocomposite Hydrogels

1.0, 2.0, or 4.0 mg of oxi-CNOs (for the non-covalent hydrogel) or 2.0 mg of Lff-PEG-CNOs (for the covalent hydrogel) were added to 1.0 mL of a solution containing 5.0 mg of Lff in alkaline sodium phosphate (0.1 M, pH 11.8) and sonicated for 15 min. Then, an equal volume of a mildly acidic sodium phosphate buffer (0.1 M, pH 5.8) was added to reach a pH of 7.4 and trigger the peptide self-assembly and hydrogelation.

### 2.18. Zeta (ζ) Potential Measurements

Oxi-CNOs and Lff-PEG-CNOs were dispersed in the gel-precursor solutions with or without Lff tripeptide at the concentration of 0.2 μg/mL and placed inside the cuvettes for the measurement of ζ-potential in a Malvern Zetasizer Nano (Alfatest, Milan, Italy). Measurements were performed at 25 °C and average values were calculated with Excel (*n* = 3).

### 2.19. CNOs’ Release Study Form the Nanocomposite Hydrogels

Non-covalent hydrogels (0.3 mL) with oxi-CNOs (1.0 mg/mL, 0.1 mg/mL or 10 μg/mL) and Lff tripeptide were prepared as described in Section 2.17 inside 15-mL Falcon tubes and left to settle overnight. The following morning, 5 mL of PBS (0.137 M NaCl, 2.7 mM KCl, 10 mM phosphate buffer, pH 7.4, prepared using PBS tablets from Sigma-Aldrich, Milan, Italy) were carefully poured on top without disrupting the gels and placed in an incubator at 37 °C, with mild shaking (60 rpm). Samples measuring 0.15 mL from three independent replicas of each condition were carefully analyzed at different timepoints for absorbance at 800 nm using a TECAN 1000 Infinite Pro microplate reader at room temperature, and then they were immediately added back to the solutions. A calibration curve was obtained from oxi-CNOs in PBS at different concentrations, with each condition repeated three times. Results were analyzed with Excel.

### 2.20. Water-Drop Contact Angle Measurements

Non-covalent hydrogels (70 μL) were prepared with (1.0 mg/mL, 0.1 mg/mL or 10 μg/mL) or without oxi-CNOs as described in Section 2.17 in the microwells of a Ibidi μ-slide Angiogenesis uncoated (Ibidi, Gräfelfing, Germany) so that the gel filled perfectly the well to the very top. The gels were left to settle for 30 min, then a 15 μL-drop of milliQ water was placed on top and immediately photographed against a black background. Three independent replicas were prepared for each condition, and contact angles measured with ImageJ2 software. Average values and standard deviations were calculated with Excel.

### 2.21. Live/Dead Cell Imaging Assay

The live/dead assay was performed according to a modified protocol [69]. Gels (20 μL) were prepared without or with oxi-CNOs (0.1, 1.0, and 10 μg/mL oxi-CNOs) as described in Section 2.17 inside the inner wells of an Ibidi μ-slide angiogenesis uncoated (Ibidi, Gräfelfing, Germany) and left to settle at room temperature for 1 h, of which 15 min under UV-irradiation using the UV-lamp of the cell-culture fumehood. Then, 30 μL of medium (DMEM + 10% fetal serum albumin, +2% antimycotic and antibiotic from Gibco, ThermoFisher, Milan, Italy) were gently added into the outer wells. After 1 h, the medium was exchanged with fresh medium with 10,000/well NIH3T3 fibroblast cells (Life Sciences Dept., University of Trieste, Italy) and cultured at 37 °C, 5% CO_2_ for 24 h, by handling the slides according to the manufacturer’s instructions. Acridine orange (5 μL/well of a 20 μM solution in 50 mM PBS) and propidium iodide (5 μL/well of a 30 μM solution in 50 mM PBS) were added in each well. Cells were imaged on a Leica microscope (DFC450C, software LASV4.13, Leica, Milan, Italy) with green and red filters and a 10× objective. Each condition was repeated 3 times.

### 2.22. MTT Metabolic Assay

Hydrogels (50 μL) were prepared without or with oxi-CNOs (0.1, 1.0, and 10 μg/mL oxi-CNOs) as described in Section 2.17 in 96-well microplates (tissue-culture grade, clear, round bottom) and left to settle at room temperature for 1 h, of which 15 min under UV-irradiation using the UV-lamp of the cell-culture fumehood. Phosphate buffer without peptide served as control. Then, 100 μL of medium (DMEM + 10% fetal serum albumin, +2% antimycotic and antibiotic from Gibco, ThermoFisher, Milan, Italy) were gently added. After 1 h, the medium was exchanged with fresh medium with 10,000/well NIH3T3 fibroblast cells (Life Sciences Dept., University of Trieste, Italy) and cultured at 37 °C, 5 % CO_2_ for 24 h. Next, 10 μL of the MTT labeling reagent (Sigma, Milan, Italy, 0.5 mg mL^–1^) were added, and the microplate was incubated for 4 h in a humidified atmosphere (37 °C, 5% CO_2_). Next, 100 μL of the solubilization solution for formazan crystals (4 mM HCl + 0.1% IGEPAL in isopropanol) was added to each well, and the microplate was kept at room temperature under shaking (Rocker-Shaker MR-12 Biosan, Vetrotecnica, Padova, Italy) for 30 min. To solubilize the gels, 7 μL methanol were added to each well, and samples were pipetted up and down to obtain a homogeneous solution. After visual inspection for complete solubilization of the purple formazan crystals, the absorbance was read at 570 nm with a reference wavelength of 690 nm (light scattering and oxi-CNOs absorbance) using a microplate reader (TECAN Infinite M1000 Pro, Milan, Italy). Each condition was repeated in 6 replicas and statistical analysis performed in Excel as described in Section 2.23.

### 2.23. Statistical Analysis

Statistical analysis was performed using the t test formula in Excel over 6 replicas for each tested condition. *p* < 0.05 was used as reference value for statistical significance.

## 3. Results

### 3.1. Design Strategies for the Nanocomposite Supramolecular Peptide Hydrogels with CNOs

**Figure 1:**
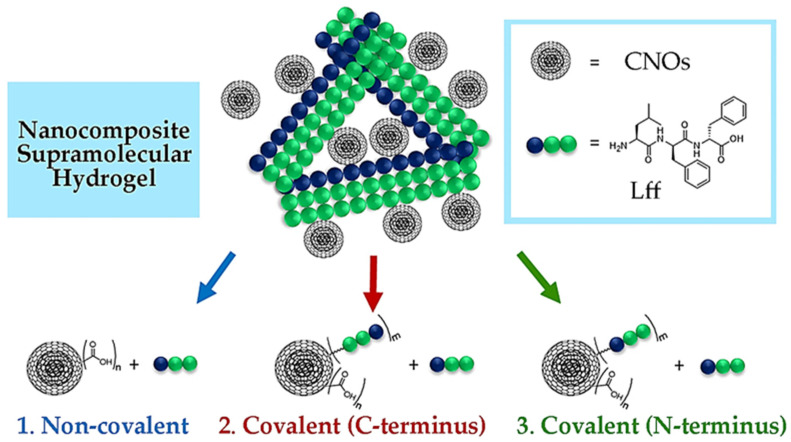
Nanocomposite supramolecular hydrogels from CNOs and the self-assembling tripeptide (Lff) were envisaged through three strategies.

The tripeptide L-Leu-D-Phe-D-Phe (Lff) was recently reported for its self-assembling ability in phosphate-buffered solutions [68]. The tripeptide was first dissolved at an alkaline pH in its anionic form, ensuring intermolecular repulsion. Subsequent lowering to neutral pH values led to the zwitterionic peptide form, which could self-assemble into hydrogels. Key intermolecular interactions included ionic bridges between the charged termini, H-bonding between amide bonds, and steric zippers holding together the hydrophobic sidechains. The inclusion of CNOs into the peptide hydrogel was investigated through three different strategies (Figure 1):

1. Non-covalent approach (i.e., by mixing oxi-CNOs and Lff);
2. C-terminal covalent approach (i.e., covalently linking Lff through the C-terminus to the CNOs, and then mixing with free peptide for co-assembly);
3. N-terminal covalent approach (i.e., covalently linking Lff through the N-terminus to the CNOs, and then mixing with free peptide for co-assembly).

The choice for oxi-CNOs in the first approach was made to favor their dispersibility in phosphate buffer, while in the other two cases, the oxi-CNOs were further functionalized with the peptide by using a PEG linker to ensure conformational flexibility of the self-assembling moiety. The CNOs were envisaged to act as reticulating agents either through simple hydrophobic interactions with the peptide (route 1) or through the intercalation of the bound peptide on the CNO surface within the stacks of free peptide (routes 2 and 3). For the three strategies, the Lff tripeptide and its derivatives were synthesized by solid phase, purified, and characterized by LC-MS, ^1^H-NMR, and ^13^C-NMR (Figures S1–S13).

### 3.2. Oxi-CNOs and Amino-PEG-CNOs Preparation and Characterization

**Figure 2:**
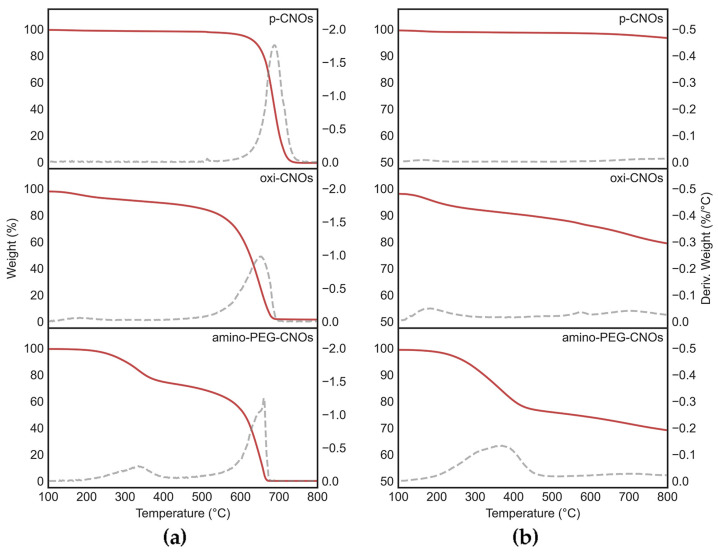
TGA data in (**a**) air and (**b**) nitrogen atmosphere of p-CNOs, oxi-CNOs, and amino-PEG-CNOs. Weight loss (red lines) and its first derivative over temperature (grey dashed lines) were consistent with the covalent functionalization of CNOs.

Pristine CNOs (p-CNOs) were firstly oxidized using an established procedure to introduce COOH groups on their surface and render them more easily dispersible in aqueous conditions. Next, they were functionalized with a PEG linker to favor peptide covalent attachment [61]. Characterization was performed by TGA under an air and a nitrogen atmosphere, by Raman and ATR-FTIR spectroscopy, and through TEM imaging. TGA data (Figure 2) confirmed the high purity of CNOs (<1.5% residual weight at 800 °C in all cases), as well as the successful preparation of oxi-CNOs (9.4–12.9% weight loss at 500 °C in nitrogen and air, respectively) and amino-PEG-CNOs (23.4–30.3% weight loss at 500 °C in nitrogen and air, respectively), relative to p-CNOs (1.4–1.9% weight loss at 500 °C in nitrogen and air, respectively). As a result of the partial conversion of some sp^2^ C atoms to sp^3^ with the consequent introduction of defects in the structure, the CNOs decomposition temperature decreased from 688 °C (p-CNOs) to 653 °C (oxi-CNOs) and 649 °C (amino-PEG-CNOs). 

**Figure 3:**
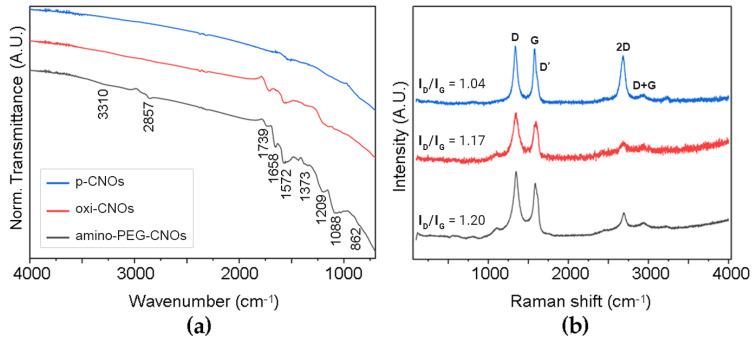
Spectra of p-CNOs, oxi-CNOs and amino-PEG-CNOs from analysis by (**a**) ATR-FTIR and (**b**) Raman spectroscopy.

ATR-FTIR spectra of all samples (Figure 3a) showed a band at 1572 cm^−1^ that originated from the stretching modes of sp^2^ carbon atoms. As expected, the oxi-CNO spectra presented two signals at 1739 and 1209 cm^−1^ from C=O and C-OH stretching modes, respectively. These same signals were found in the spectra of amino-PEG-CNOs, indicating that not all surface carboxylic acid groups were PEGylated. Free carboxylic groups on the CNO surface are desirable to maintain sufficient hydrophilicity for the material to be well dispersed in phosphate buffer. Nevertheless, the successful functionalization was confirmed by the fact that linker signals were predominant in the spectra of amino-PEG-CNOs, in particular those at 3310 cm^−1^ and 1658 cm^−1^ assigned to the N-H and C=O stretching of amide groups, respectively, together with signals arising from the linker chain (2857, 1373, 1088 and 862 cm^−1^). Likewise, Raman spectra (Figure 3b) revealed an increase in sp^3^ defects upon functionalization, with I_D_/I_G_ increasing from 1.04 ± 0.08 of p-CNOs to 1.17 ± 0.08 of oxi-CNOs and 1.20 ± 0.07 of amino-PEG-CNOs. The significant increase in the I_D_/I_G_ ratio between p-CNOs and oxi-CNOs occurred due to the conversion of surface sp^2^ carbon atoms to sp^3^ carbon atoms during oxidation. In contrast, a significant increase in the I_D_/I_G_ ratio between oxi-CNOs and amino-PEG-CNOs was not observed—as the PEGylation process affected the surface-bound carboxylic acid moieties of oxi-CNOs, the surface sp^2^ and sp^3^ carbon remained largely unaffected.

### 3.3. Non-Covalent Approach

**Figure 4:**
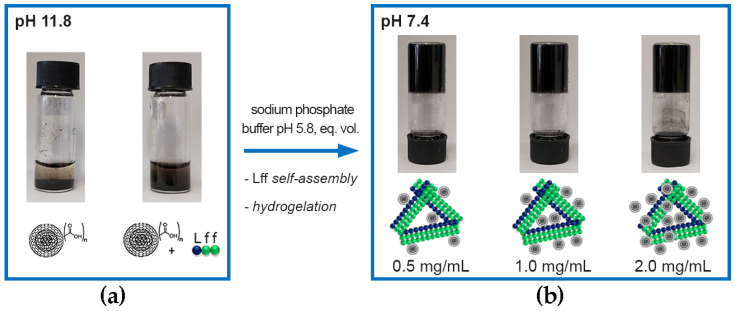
Photographs of (**a**) oxi-CNOs without (left) and with (right) the self-assembling tripeptide Lff in alkaline (pH 11.8) sodium phosphate precursor solution after 4 h post sonication (15 min) to prepare gels with 1.0 mg/mL oxi-CNOs, and (**b**) nanocomposite supramolecular hydrogels with 2.5 mg/mL of Lff and increasing loadings of oxi-CNOs (0.5, 1.0 and 2.0 mg/mL) at pH 7.4.

In the first approach, oxi-CNOs were simply mixed with the self-assembling Lff tripeptide in the alkaline (pH 11.8) phosphate solution. Oxidation of p-CNOs is an established strategy to render them more hydrophilic and, thus, dispersible in aqueous solutions. Indeed, oxi-CNOs dispersed well; however, after 20 min, they started to deposit at the bottom of the vial (Figure S14). Conversely, the presence of Lff increased the stability of the oxi-CNO dispersion; they started to sediment only after four hours (Figure 4a). This effect can be ascribed to the amphiphilic nature of the peptide that, by establishing hydrophobic interactions through its sidechains with the oxi-CNO surface, effectively shielded it from the solvent. Furthermore, solvent exposure of the hydrophilic peptide backbone facilitated H-bonding between the peptide-coated CNOs and water [68]. Subsequent lowering of the pH to 7.4 triggered gelation of the peptide (Figure 4b), also in the presence of oxi-CNOs at levels as high as 2.0 mg/mL (with 2.5 mg/mL peptide), corresponding to a remarkable 80% *w*/*w* relative to the peptide. To the best of our knowledge, this is the highest nanocarbon loading reported [68], indicating excellent compatibility between the CNOs and the peptide. In previous studies [68], nanocarbons with quasi-spherical morphology, but a more rugged surface (i.e., carbon nanohorns), could not be well-dispersed in the hydrogels and showed segregation, resulting in limited mechanical properties of the final materials. A key aspect was the incomplete coating of the nanohorns’ surface by the peptide, thus leaving their bare surface exposed towards self-aggregation. We inferred that the spherical and smoother surface of oxi-CNOs appeared to be a favorable property towards nanoparticle’s coating by the peptide, and subsequent formation of homogeneous nanocomposites.

**Figure 5:**
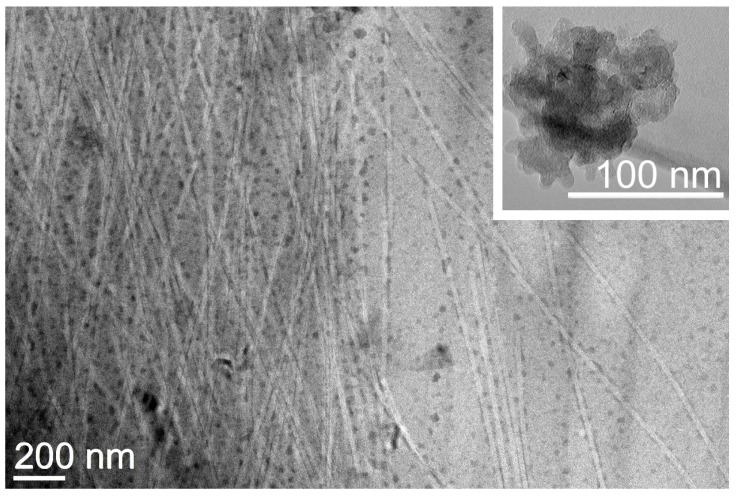
TEM micrograph of the nanocomposite supramolecular hydrogel formed by Lff in the presence of oxi-CNOs through the non-covalent approach. Inset figure shows aggregate of oxi-CNOs in the absence of the Lff peptide.

Transmission electron microscopy (TEM) imaging revealed a network of highly homogeneous fibrils of 21 ± 2 nm (*n* = 50) diameter that interacted on their surface with individual CNOs that appeared to be well-dispersed within the matrix (Figure 5). This result contrasts the TEM images of oxi-CNOs without the peptide, which instead displayed large aggregates (inset in Figure 5 and Figure S15), as is typically observed for CNMs. Overall, the TEM images confirmed the positive effect of the peptide on oxi-CNOs dispersibility, as well the positive effect of CNOs on the peptide, since their presence hindered the undesirable hierarchical assembly into bundles of fibrils and fibers of heterogeneous diameters that were observed instead in the case of the peptide alone, or with other nanocarbons [68].

**Figure 6:**
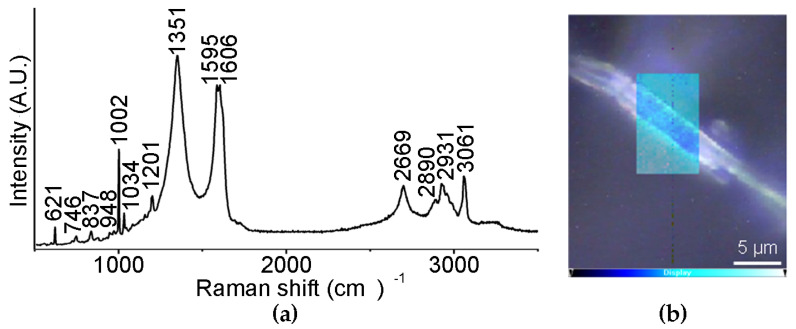
(**a**) Raman spectrum of the nanocomposite supramolecular hydrogel formed by Lff in the presence of oxi-CNOs. (**b**) Raman mapping of a non-covalent hydrogel fiber; intensity scalebar: the brighter cyan areas correspond to higher levels of oxi-CNOs.

Raman spectromicroscopy (Figure 6a) revealed the presence of the D and G bands of CNOs at 1351 and 1595 cm^−1^, respectively, as well as of the signals due to the Lff assemblies, of which the most prominent were the aromatic Phe Raman modes at 1002 cm^−1^ and 1606 cm^−1^. Interestingly, the presence of CNOs shifted other peptide signals, suggesting interactions between the two components, and in particular:

- The signal at 1037 cm^−1^ was shifted to 1034 cm^−1^ and it was attributed to the aromatic ring of Phe, being suggestive of hydrophobic interactions with oxi-CNOs
- The signal at 1207 cm^−1^ was shifted to 1201 cm^−1^ and it is in the amide III region, where signals coming from the combination of C-N stretching and N-H bending are found, suggesting differences in the H-bonding pattern due to oxi-CNOs
- The signal at 952 cm^−1^, relative to the vibrational mode of the peptide skeleton, was shifted to 948 cm^−1^, suggesting some difference in the peptide conformation upon interacting with oxi-CNOs

A Raman mapping of a hydrogel fiber (Figure 6b), based on the intensity ratio between the D band of oxi-CNOs (1351 cm^−1^) and the signal of the Phe rings at 1002 cm^−1^, showed that oxi-CNOs coated the peptide fiber, while their presence in the internal part was modest.

**Figure 7:**
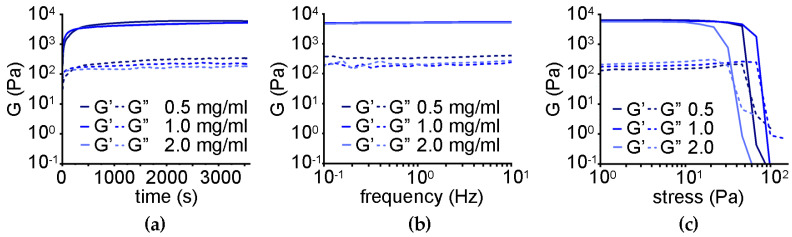
Oscillatory rheology of the nanocomposite supramolecular hydrogels obtained through the non-covalent approach. (**a**) Time sweeps. (**b**) Frequency sweeps. (**c**) Stress sweeps.

Oscillatory rheological kinetic analysis (Figure 7a) revealed rapid gelation and a significant increase in the elastic modulus G′ to a similar extent for the three different levels of oxi-CNO loading, corresponding to 5.5 ± 1.0 kPa relative to 2.0 ± 0.1 kPa of the peptide alone, and a Young modulus of 5.8 ± 1.1 kPa relative to 2.2 ± 0.1 kPa of the peptide alone [68]. Frequency sweeps (Figure 7b) confirmed the stability of the hydrogels even at the highest loading level of oxi-CNOs, with both elastic (G′) and viscous (G″) moduli being independent of the applied frequency. Finally, stress sweeps (Figure 7c) confirmed an increase in the hydrogel resistance against applied stress, ranging from 60 ± 3 to 83 ± 3 Pa for the gel-to-sol transition for gels with 0.5 and 1.0 mg/mL oxi-CNOs, respectively. Conversely, a further increase in CNOs to 2.0 mg/mL negatively impacted the rheological properties of the gel, giving the worst performance as the gel-to-sol transition occurred at 36 ± 4 Pa.

### 3.4. Covalent Approach (C-Terminus)

The coupling of the Lff peptide via the C-terminus to the amino-PEG-CNOs was attempted through several approaches, using different coupling agents, solvents, and also with the assistance of the MW-reactor. The best results were obtained with Oxyma Pure B and DIC as coupling agents in DMF; however, the thermogravimetric analysis revealed a negligible increase in the weight loss relative to the amino-PEG-CNOs precursor (ca. 1% wt.) that was not significantly different from the range of experimental error. It is possible that the *C*-terminus of Lff was too hindered due to the bulky Phe sidechain to allow its coupling to the amino-PEG-CNOs in high yields. Therefore, the peptide coupling was attempted via the N-terminus, as described in the following section.

### 3.5. Covalent Approach (N-Terminus)

To covalently attach the Lff tripeptide to oxi-CNOs, a PEG linker was first coupled to the N-terminus of the peptide by treating it just like an amino acid in the solid-phase peptide synthesis. Next, the amino-PEG-Lff was bound to the oxi-CNOs, and thermogravimetric analysis confirmed the successful functionalization (Figure S16), with a loss of 11.7% wt. at 500 °C, which was 2.3% wt. higher than that of the oxi-CNOs. This value corresponds to a loading of ca. 40 μmol/g of amino-PEG-Lff. Raman and ATR-FTIR spectra (Figures S17 and S18) did not reveal remarkable differences compared to those of oxi-CNOs, as expected for this low level of functionalization. The I_D_/I_G_ value of 1.19 ± 0.83 was unsurprisingly similar to that of oxi-CNOs since the coupling reaction did not introduce new defects on the CNO surface. While the degree of functionalization is low, it is suitable for the purpose of this work—residual carboxylic groups on the CNOs surface are necessary to ensure their dispersibility in aqueous environments, as confirmed with the gel-precursor solution (Figure S19).

**Figure 8:**
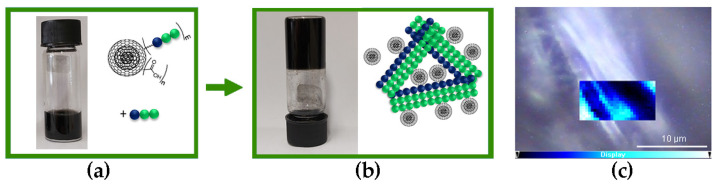
Photographs of: (**a**) Lff-PEG-CNOs (N-terminus approach) with the self-assembling tripeptide Lff in alkaline (pH 11.8) sodium-phosphate precursor solution; (**b**) nanocomposite supramolecular hydrogels with Lff-PEG-CNOs (1.0 mg/mL) and 2.5 mg/mL of Lff at pH 7.4. (**c**) Raman mapping of a hydrogel fiber; intensity scalebar: brighter cyan areas correspond to higher levels of Lff-PEG-CNOs.

The addition of free Lff peptide to Lff-PEG-CNOs resulted in a homogenous dispersion in alkaline (pH 11.8) phosphate solutions (Figure 8a), which gelled when pH was lowered to neutral (Figure 8b), in an analogous manner as previously observed for the non-covalent approach (Figure 4). Raman mapping (Figure 8c) also led to analogous results as those for the non-covalent approach (Figure 6b), with Lff-PEG-CNOs being located mainly on the surface of the peptide fibers, as opposed to their interior. However, the presence of heterogeneously distributed bright areas could indicate CNO aggregation, as revealed by TEM imaging (vide infra). 

**Figure 9:**
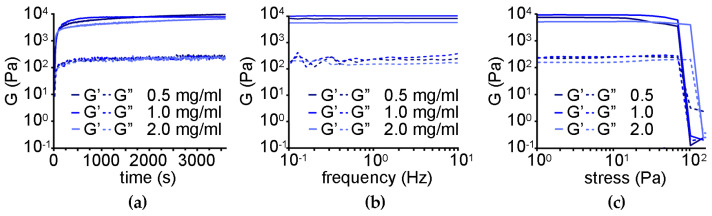
Oscillatory rheology of the nanocomposite supramolecular hydrogels obtained through the covalent approach. (**a**) Time sweeps. (**b**) Frequency sweeps. (**c**) Stress sweeps.

Oscillatory rheology revealed similar gelation kinetics relative to the nanocomposite supramolecular hydrogel obtained through the non-covalent approach (Figure 9a). An increase in the elastic modulus G’ to 9.7 ± 2.0 kPa was found for the gels with 0.5 or 1.0 mg/mL CNO loading levels, corresponding to a Young modulus of 10.0 ± 2.1 kPa. Conversely, a further increase to 2.0 mg/mL of CNOs corresponded to a decrease in G′ to 6.6 ± 1.0 kPa, suggesting that of the three tested levels, 1.0 mg/mL was the optimal option. Frequency sweeps confirmed hydrogels’ stability in all cases (Figure 9b). Finally, stress sweeps revealed an increased resistance against applied stress, with gel-to-sol transitions occurring at 98 ± 16 Pa for the gels with either 0.5 or 1.0 mg/mL CNOs, and, surprisingly, this value was further raised to 135 ± 5 Pa for the gel with 2.0 mg/mL CNOs (Figure 9c). Overall, this dataset suggested different nanomorphological features of the gelling fibers relative to the non-covalent gel, giving scope for TEM imaging.

**Figure 10:**
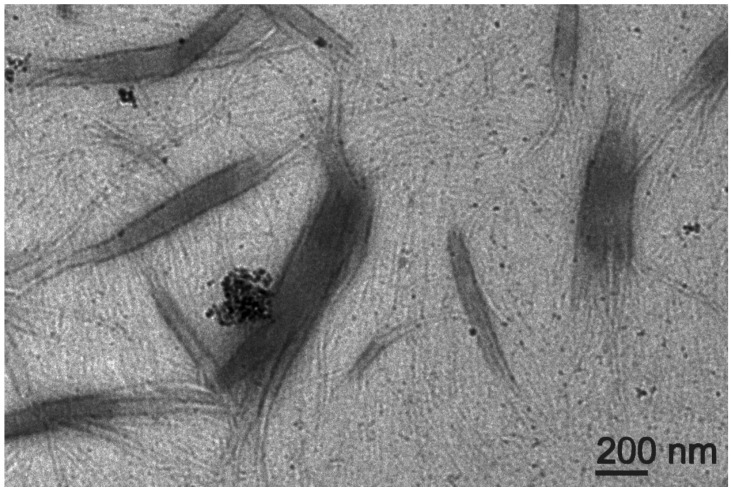
TEM micrograph of the nanocomposite supramolecular hydrogel formed by Lff in the presence of Lff-PEG-CNOs through the covalent approach.

TEM micrographs revealed that the gel obtained through the covalent approach displayed not only a dense network of fibrils—analogously to the non-covalent gel (Figure 5)—but also multiple connecting points where fibrils were grouped in branching bundles as thick and long as a few hundreds of nanometers (Figure 10). CNOs were generally well dispersed in the material, although aggregates were present too, especially in areas where multiple fibrils were branching out in different directions (Figure 10, Figures S20 and S21). On the one hand, these observations confirmed that the covalent approach successfully anchored the Lff peptides onto the CNO surface to act as reticulating agents between fibrils. On the other hand, the presence of self-assembling peptides on the surface of CNOs could also drive undesired aggregation of CNOs and peptides, overall leading to a heterogenous material. Indeed, when the peptide was added to the gel precursor solution with Lff-PEG-CNOs, sedimentation into aggregates started to occur after 10 min (Figure S22), in contrast with the four hours for the non-covalent sample (Figure 4a).

### 3.6. Nanocomposites’ Stability and Cytocompatibility for Biological Applications

The data above indicated that the nanocomposites formed with the non-covalent approach were more homogeneous, without aggregates, and with better coating of the oxi-CNOs by the peptide, relative to those formed through the covalent approach. The colloidal stability of the CNOs in the gel-precursor solutions was further probed by zeta (ζ) potential measurements to confirm these findings. This parameter is defined as the potential at the slipping plane, which is related to the surface charge of the CNOs and the chemical composition of the local environment. It provides an accurate estimation of the aggregation tendency of the nanoparticles, that is significantly reduced by electrostatic-charge repulsion for values that exceed +30 or −30 mV, and that provide moderate colloidal stability [70]. Accordingly, the larger the absolute value of the ζ-potential, the more stable are the nanoparticles. The oxi-CNOs displayed a ζ-potential of −29.9 ± 0.9 mV, as expected due to the presence of the acidic groups on their surface. Addition of the free peptide, which is present as an anion in the alkaline buffer solution, significantly shifted the value to −51.2 ± 1.7, while samples with Lff-PEG-CNOs with free peptide displayed a ζ-potential of −33.9 ± 0.9 mV. We inferred that the oxi-CNOs had more peptide that was coating the nanoparticles, relative to Lff-PEG-CNOs. This data is consistent with the increased colloidal stability of oxi-CNOs in the gel-precursor solutions, as discussed above.

In light of these findings, the non-covalent approach was thus selected for subsequent cytocompatibility studies. Firstly, the stability of the nanocomposite hydrogels was tested at physiological conditions, i.e., in phosphate-buffered-saline (PBS) solutions at 37 °C, with mild agitation. Different oxi-CNO loading levels were probed, ranging from 10 μg/mL to 1 mg/mL. Lower levels were not tested due to detection limits of the experimental setup, which used visible-light absorbance to monitor oxi-CNOs release from the biomaterial scaffold. Over the course of 24 h no release of oxi-CNOs was noted for the hydrogels loaded with 10 or 100 μg/mL, and <1% oxi-CNOs were released from the nanocomposite with the highest loading (see Supplementary File, Figure S23). The water-drop contact angle for the hydrogels indicated good hydrophilicity, as expected, and ranged from 33 ± 2° for the peptide alone, to 39 ± 1°, 42 ± 2° and 44 ± 1° for the hydrogels with 10 μg/mL, 100 μg/mL, and 1 mg/mL oxi-CNOs, respectively. All the contact angles for the different conditions were statistically significantly different (*p* < 0.005), and increased with higher loadings of oxi-CNOs, indicating a slight increase in hydrophobicity.

**Figure 11:**
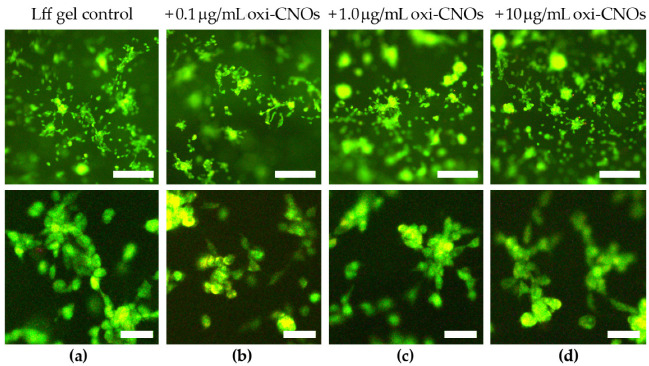
Live (green)/dead (red) fluorescence staining of fibroblasts grown in contact with the peptide hydrogel without (**a**) or with (**b**–**d**) oxi-CNOs. Scalebars represent 50 μm (top panels) or 10 μm (bottom panels).

Having established the biomaterials’ stability, their cytocompatibility was probed in vitro. The hydrogels were prepared with three different loading levels of oxi-CNOs that could ensure sufficient optical transparency for light microscopy, i.e., 0.1, 1.0, and 10 μg/mL. Peptide hydrogels without oxi-CNOs served as controls for the 3D scaffold. Fibroblast cells seeded on the hydrogels adhered to, and proliferated well into, all the biomaterials, with no significant difference between the tested conditions (Figure 11). Furthermore, cell penetration into the soft scaffold was noted, with cells being located at different levels of depth, as can be seen by out-of-focus fluorescence. Live/dead staining confirmed presence of healthy cells in high numbers and with spread morphology, suggesting good adherence to the soft scaffold.

**Figure 12:**
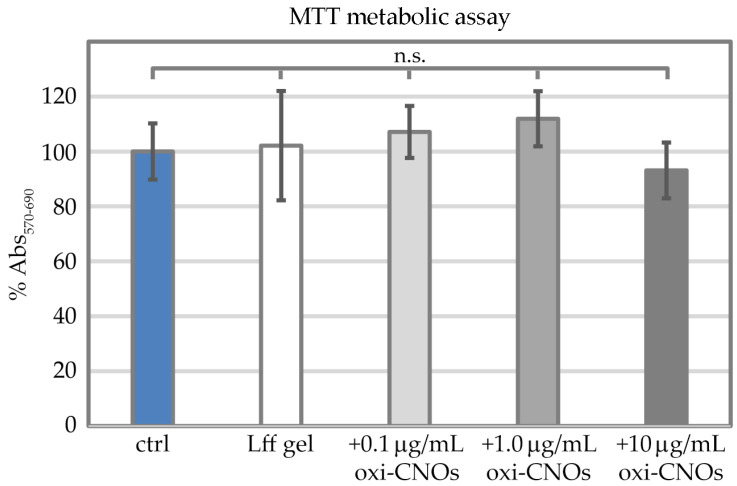
MTT metabolic assay on fibroblast cells grown on the plastic control (ctrl), the peptide gel without (Lff gel) or with increasing concentrations of oxi-CNOs (+0.1, +1.0, or +10 μg/mL oxi-CNOs). No statistically significant (n.s.) difference was found between tested conditions (*p* > 0.05).

Metabolic assays were performed to obtain another quantitative measure of cell proliferation in the presence of the nanocomposite hydrogels (Figure 12). To this end, 1-(4,5-dimethylthiazol-2-yl)-3,5-diphenylformazan (MTT) was used as an indicator of mitochondrial activity. Fibroblast cells demonstrated analogous levels of metabolic activity across the tested conditions (Figure 12), i.e., without gel, with the peptide gel without oxi-CNOs, or with the peptide gels with oxi-CNOs (0.1, 1.0, and 10 μg/mL). No statistically significant difference was found amongst samples.

## 4. Conclusions

In conclusion, this work demonstrated that oxi-CNOs and the self-assembling tripeptide Lff are highly compatible with attaining nanocomposite supramolecular hydrogels at physiological conditions (i.e., phosphate buffer at pH 7.4) for biological use. The presence of oxi-CNOs also at the remarkably high loading of 80% *w*/*w* relative to the peptide did not interfere with peptide self-organization into fibrils, surpassing all the other CNMs tested thus far [68]. Furthermore, oxi-CNOs hindered the bundling of fibrils into fibers, yielding a highly homogeneous nanofibrillar gel. Another advantage gained by combining Lff with CNOs was due to the peptide’s amphiphilic nature that prolonged the dispersibility of oxi-CNOs in sodium-phosphate solutions by several hours and increased their ζ-potential in the gel-precursor solutions from −30 mV to −51 mV, thus reducing their aggregation propensity and increasing their colloidal stability.

The nanocomposite supramolecular hydrogels obtained through the non-covalent approach, i.e., by simply mixing the oxi-CNOs with the Lff tripeptide, were thoroughly characterized by spectroscopic, microscopic, and thermogravimetric analyses and demonstrated interactions between the two components as well as the absence of oxi-CNO aggregates within the hydrogel matrix that ensured a homogenous dispersion of the nanocarbon. These hydrogels displayed good hydrophilicity as required for biomaterial scaffolds, with contact angles in the range of 39–44°, depending on the loading level of oxi-CNOs. These biomaterials also displayed excellent stability at physiological conditions, with negligible release of oxi-CNOs within 24 h, and excellent cytocompatibility in vitro.

The covalent binding of the Lff tripeptide with the oxi-CNOs was also attempted, but very low levels of peptide loading were obtained, and the material displayed improved rheological properties at the expense of CNO aggregation. Indeed, anchoring of the self-assembling tripeptide on the CNOs had a two-fold outcome: on the one hand, it acted as a reticulating agent by providing connection points between fibrils, but on the other hand, it also caused undesirable aggregation leading to a heterogeneous material. All in all, the non-covalent approach was the best strategy, especially considering its simplicity. This work opened the way to developing nanocomposite hydrogel biomaterials based on simple tripeptides and oxi-CNOs for advanced applications, such as targeted drug delivery and bioimaging.

## Data Availability

Data is available in Supplementary Materials File and from the authors upon reasonable request.

## Supplementary Materials

The following supporting information can be downloaded at: https://www.mdpi.com/article/10.3390/nano13010172/s1, spectroscopic data of Lff and its derivatives (Figures S1–S13), photograph of the CNOs dispersion in the gel-precursor solution (Figure S14), TEM image of CNOs (Figure S15), TGA data (Figure S16), Raman data (Figure S17), ATR-FTIR data (Figure S18), photographs of the Lff-PEG-CNO dispersion prior to peptide gelation (Figure S19), TEM images of the covalent gel (Figures S20–S21), photographs of the Lff-PEG-CNOs dispersion in the gel-precursor solution with Lff (Figure S22), oxi-CNOs release data (Figure S23).
