# Supplementary material for: Supramolecular Hydrogels from a Tripeptide and Carbon Nano-Onions for Biological Applications
Source: Nanomaterials (Basel). 2022 Dec 30;13(1):172. doi: 10.3390/nano13010172 (PMC9824889; doi:10.3390/nano13010172)
Supplement: Supplementary file 1 [file nanomaterials-13-00172-s001.zip › nanomaterials-2100274-supplementary.pdf]

Supporting Information

Supramolecular Hydrogels from a Tripeptide and Carbon Nano-Onions for Biological Applications

[1] Chemical and Pharmaceutical Sciences Department, University of Trieste, 34127 Trieste, Italy
[2] School of Chemical Sciences, Faculty of Science & Health, Dublin City University, D09 E432 Dublin, Ireland
[3] Department for Materials Synthesis, Jožef Stefan Institute, 1000 Ljubljana, Slovenia
[4] Department of Pharmaceutical Technology, Faculty of Pharmacy, University of Ljubljana, 1000 Ljubljana, Slovenia
[5] Department of Life Sciences, University of Trieste, 34127 Trieste, Italy
* Correspondence: smarchesan@units.it (S.M.); silvia.giordani@dcu.ie (S.G.)

Table of Contents

1. Spectroscopic data for Lff…………….……………………………………………… 2
2. Spectroscopic data for Fmoc-Lff…………………………………………………… 5
3. Spectroscopic data for amino-PEG-Lff………...……………………………………….. 8
4. Photographs of oxi-CNOs dispersed in the gel-precursor solution …….……….. 11
5. TEM image of oxi-CNOs……………………………………………………………….. 12
6. Characterization of Lff-PEG-CNOs…………………………………………………... 12
7. Photographs of Lff-PEG-CNOs dispersed in the gel-precursor solution……………. 14
8. TEM images of covalent gel with Lff-PEG-CNOs……………………………………… 14
9. Photographs of Lff-PEG-CNOs dispersions in the gel-precursor solution with the Lff tripeptide……………………………………………………………………………….. 15
10. Oxi-CNOs release study………………………………………………………………. 16

1. Spectroscopic data for Lff

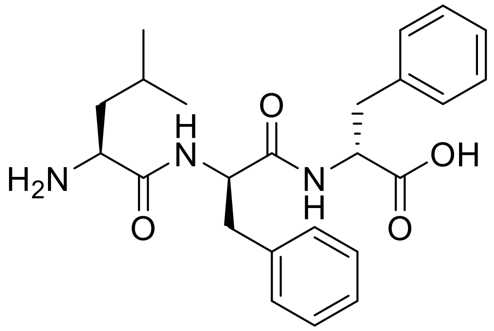

^{1}H-NMR (400 MHz, DMSO-*d_6*): δ (ppm) 12.70 (s (br), COOH), 8.73 (d, *J* = 8.5 Hz, 1H, NH), 8.62 (d, *J* = 8.6 Hz, 1H, NH), 7.98 (s (br), 2H, NH_2), 7.32–7.16 (m, 10H, Ar), 4.72 (ddd, *J* = 10.6, 8.6, 4.5 Hz, 1H, αCH), 4.47 (m, 1H, αCH), 3.64 (m, 1H, αCH), 3.11 (dd, *J* = 4.4, 13.7 Hz, 1H, βCH_2), 2.95 (dd, *J* = 6.7, 13.7 Hz, 1H, βCH_2), 2.78 (dd, *J* = 10.7, 13.6 Hz, 1H, βCH_2), 2.65 (dd, *J* = 7.8, 13.7 Hz, 1H, βCH_2), 1.16 – 1.06 (m, 3H, γCH, βCH_2), 0.68 (dd, *J* = 5.7 Hz, 6H, δCH_3). **^{13}C NMR** (101 MHz, DMSO-*d_6*): δ (ppm) 172.7, 171.2, 168.7 (3xCO); 137.5, 137.4, 129.3, 129.1, 128.3, 128.0, 126.5, 126.3 (Ar); 53.7, 53.6, 50.6 (3xαC); 40.3, 38.1, 36.6 (3xβC); 23.1 (γC); 22.5, 21.7 (2xδC). **MS (ESI):** m/z 426.1 $(M+H)^+$, $C_{24}H_{31}N_3O_4$ requires 425.2.

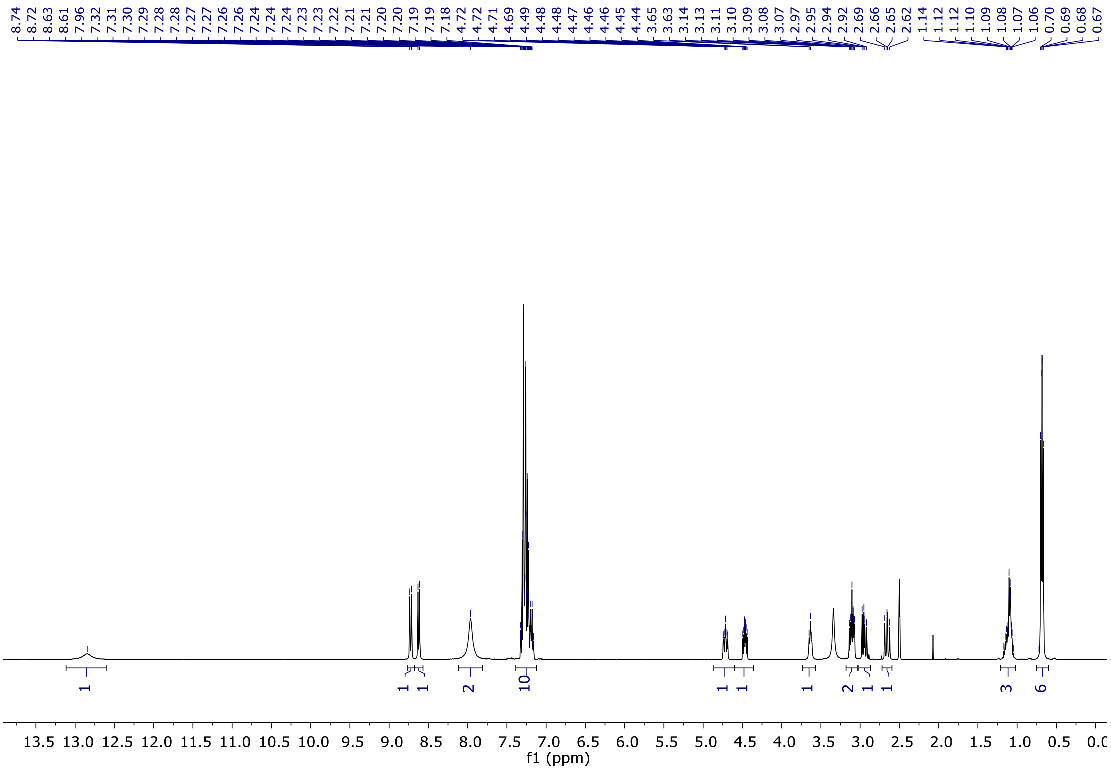

Figure S1. 400 MHz ^{1}H-NMR spectrum of Lff in DMSO-d_6

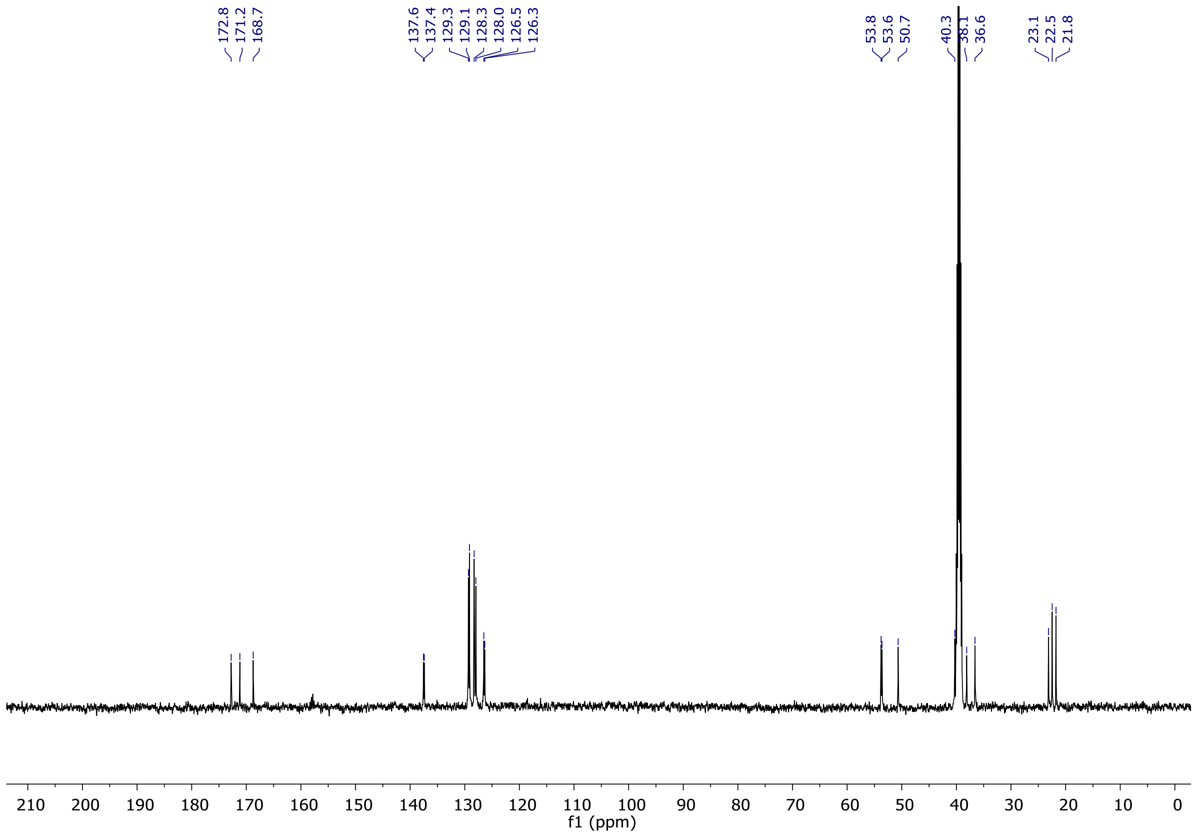

Figure S2. 400 MHz ^{13}C-NMR spectrum of Lff DMSO-d_6

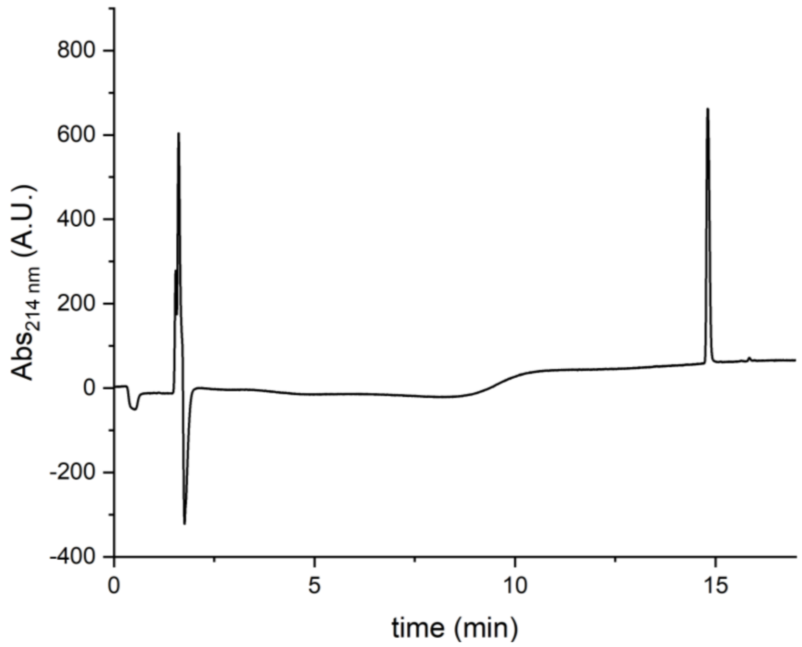

Figure S3. HPLC trace of purified Lff

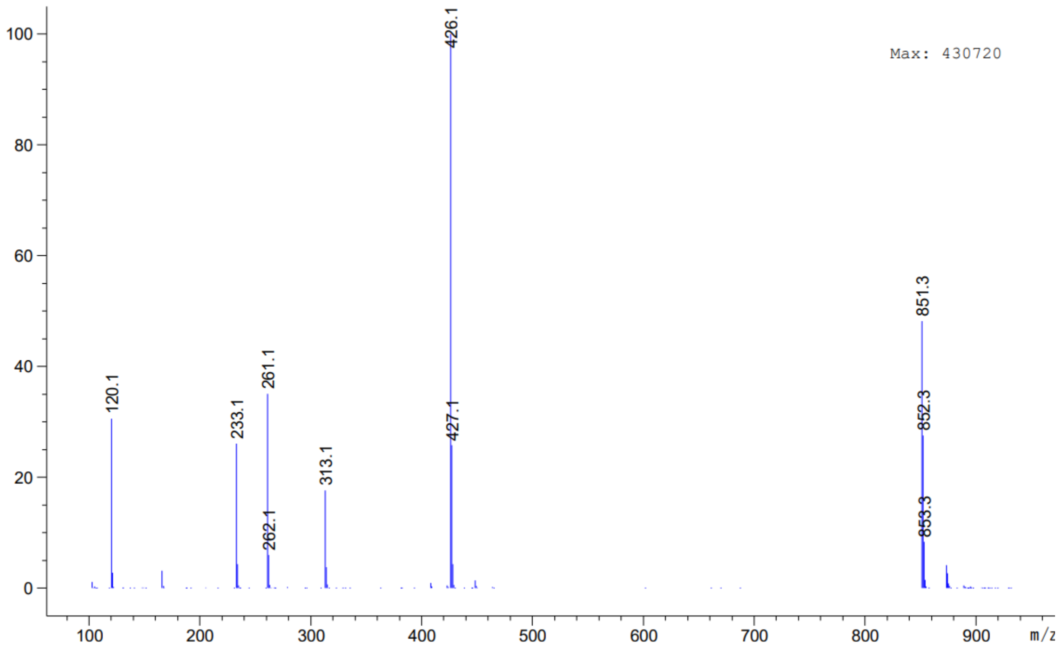

Figure S4. ESI-MS spectrum of Lff in positive ion mode

2. Spectroscopic data for Fmoc-Lff

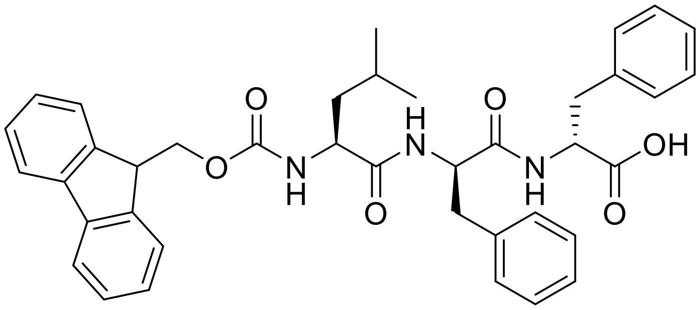

^{1}**H NMR** (400 MHz, DMSO-d_6): δ (ppm) 12.70 (s, 1H, COOH), 8.25 (d, *J* = 7.8 Hz, 1H, NH), 8.21 (d, *J* = 8.7 Hz, 1H, NH), 7.88 (d, *J* = 7.65, 2H, Ar^{Fmoc}), 7.71 (m, 2H, Ar^{Fmoc}), 7.74 – 7.67 (m, 2H, Ar^{Fmoc}), 7.46 (d, *J* = 7.9 Hz, 1H, NH), 7.41 - 7.31 (m, 2H, Ar^{Fmoc}), 7.28 – 7.10 (m, 10H, Ar^{Phe}), 4.53 (ddd, *J* = 10.8, 8.7, 3.7 Hz, 1H, αCH), 4.45 (td, *J* = 8.2, 5.7 Hz, 1H, αCH), 4.30 – 4.12 (m, 3H, CH^{Fmoc}, CH_2^{Fmoc}), 3.97 (td, *J* = 8.7, 6.0 Hz, 1H, αCH), 3.13 – 2.89 (m, 3H, βCH_2), 2.67 (dd, *J* = 13.7, 10.9 Hz, 1H, βCH_2), 1.31 – 1.02 (m, 3H, βCH_2, γCH), 0.74 (m, 6H, δCH_3). ^{13}**C NMR** (101 MHz, DMSO-d_6): δ (ppm) 172.6, 172.1, 171.2 (3xCO), 156.0, 143.9, 143.7, 140.7, 137.8, 137.4, 129.3, 129.1, 128.2, 127.9, 127.6, 127.0, 126.4, 126.1, 125.3, 120.1 (Ar), 65.7 (CH_2^{Fmoc}), 53.6, 53.1 (2xαC), 46.6 (CH^{Fmoc}), 40.6, 37.3, 36.7 (3xβC), 23.9 (γC), 22.8, 21.7 (2xδC). **MS (ESI):** m/z 670.3 $(M+Na)^+$, $C_{39}H_{41}N_3O_6$ requires 647.3.

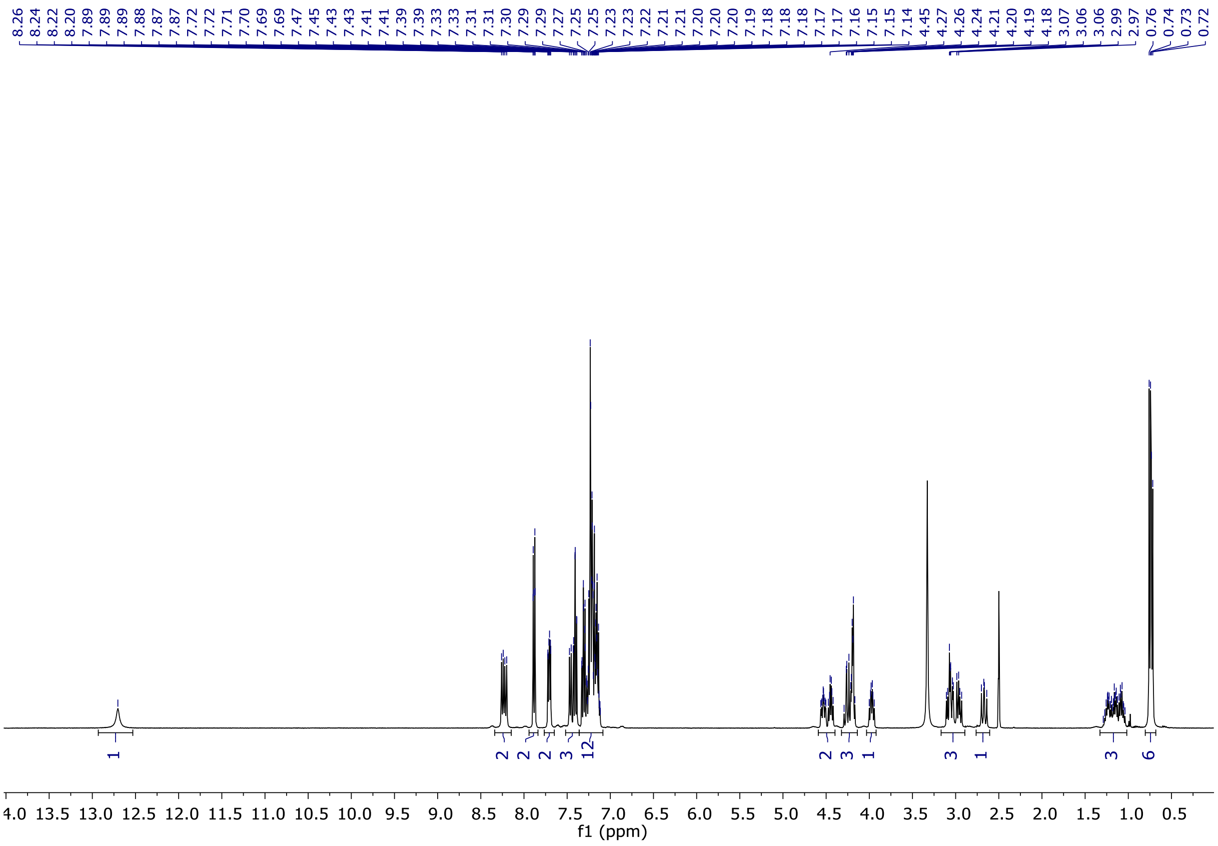

Figure S5. 400 MHz ^{1}H-NMR spectrum of Fmoc-Lff in DMSO-d_6

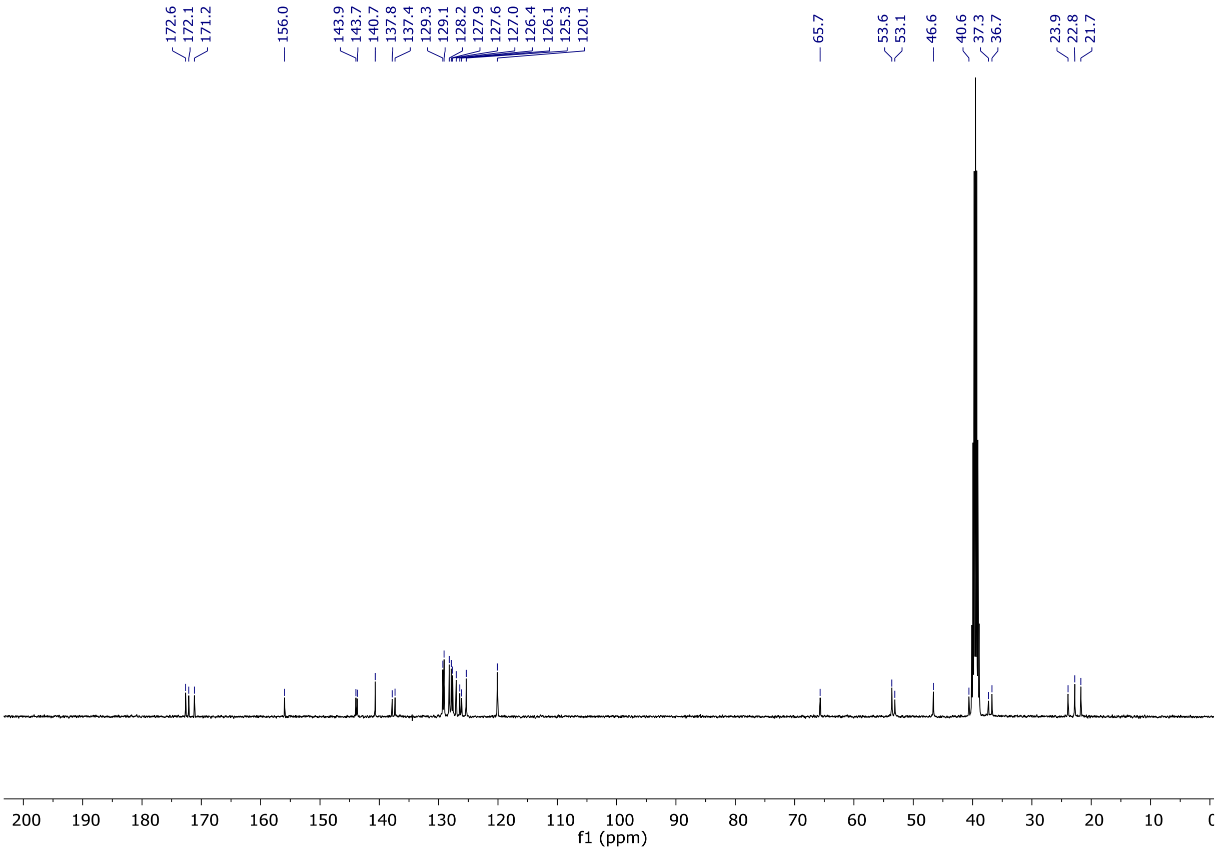

Figure S6. 400 MHz ^{13}C-NMR spectrum of Fmoc-Lff in DMSO-d_6

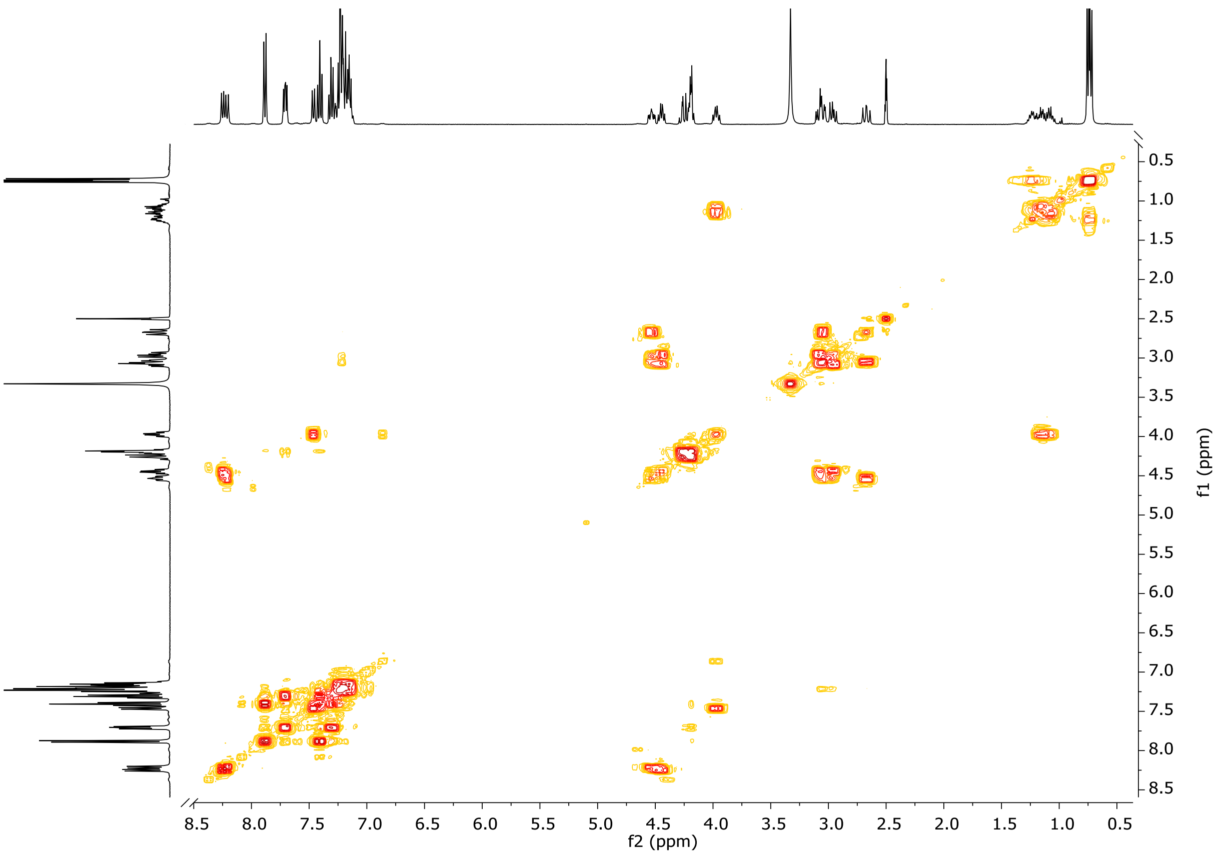

Figure S7. 400 MHz $^{1}H,^{1}H$ COSY spectrum of Fmoc-Lff in DMSO-d_6

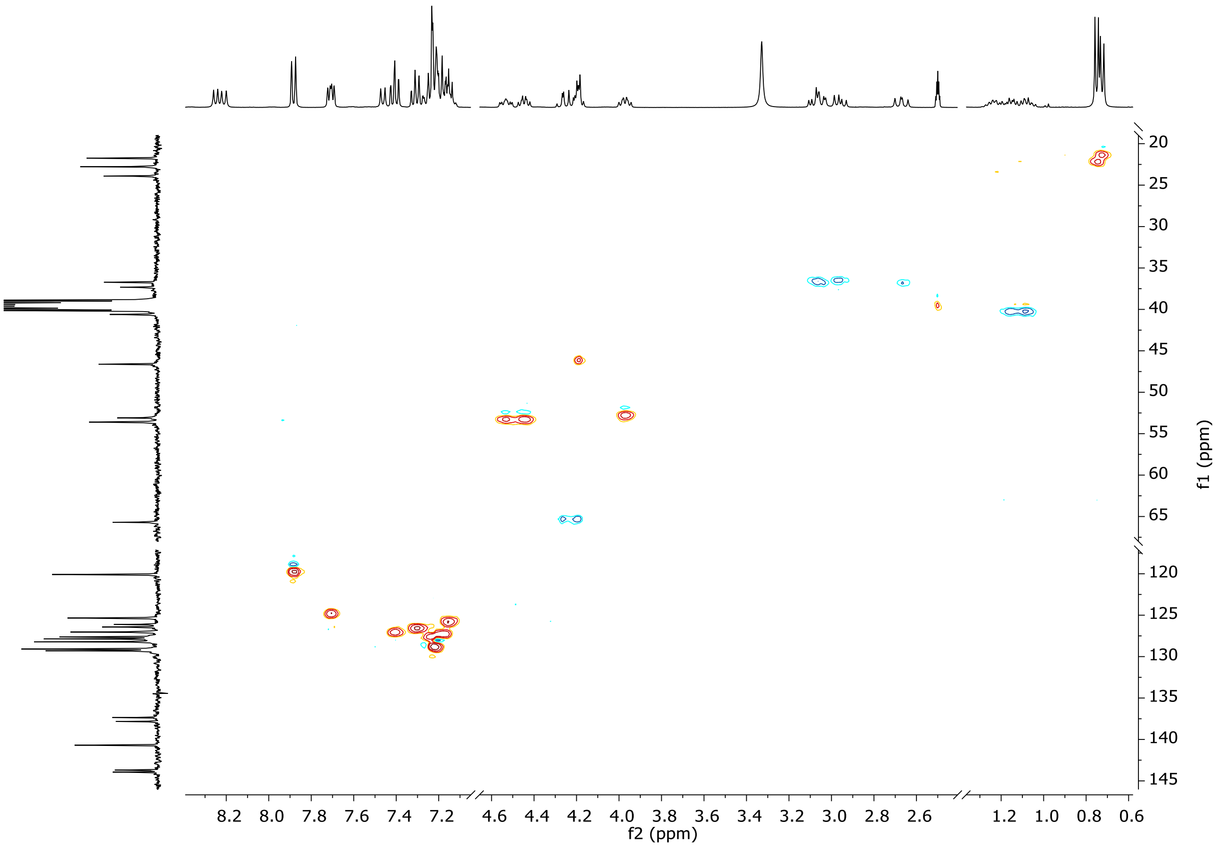

Figure S8. 400 MHz $^{1}H,^{13}C$ HSQC spectrum of Fmoc-Lff in DMSO-d_6

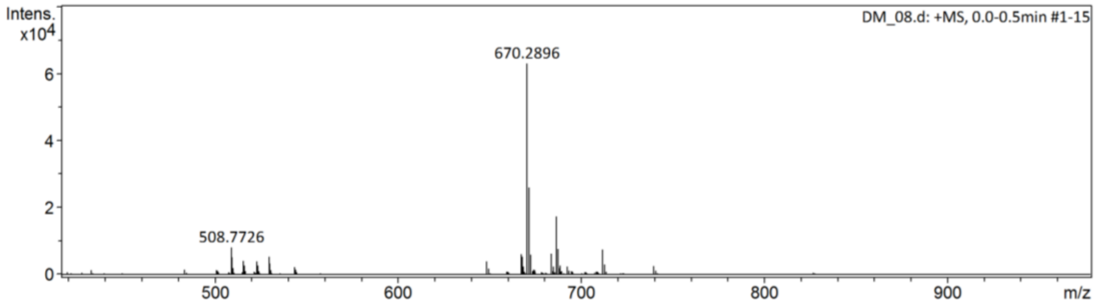

Figure S9. ESI-MS spectrum of Fmoc-Lff in positive ion mode

3. Spectroscopic data for amino-PEG-Lff

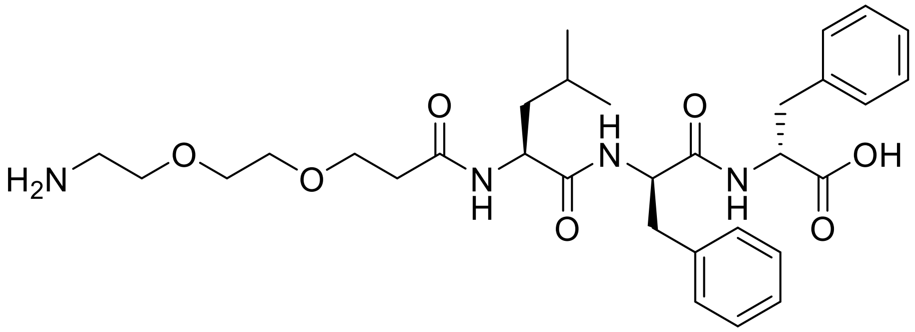

^{1}H NMR (400 MHz, DMSO-d_6): δ (ppm) 8.26 (d, J = 8.7 Hz, 1H, NH), 8.18 (d, J = 7.6 Hz, 1H, NH), 7.92 (d, J = 7.9 Hz, 1H, NH), 7.40 – 7.09 (m, 10H, Ar), 4.48 (m, 1H, αCH), 4.37 (td, J = 8.0, 5.4 Hz, 1H, αCH), 4.24 (m, 1H, αCH), 3.61 – 3.44 (m, 8H, CH_2^{PEG}), 3.13 – 2.88 (m, 5H, βCH_2, CH_2^{PEG}), 2.65 (dd, J = 13.8, 11.2 Hz, 1H, βCH_2), 2.34 (m, 2H, CH_2^{PEG}), 1.19 – 1.01 (m, 3H, βCH_2,γCH), 0.70 (m, 6H, δCH_3). **^{13}C NMR** (101 MHz, DMSO-d_6): δ (ppm) 172.8, 171.8, 171.1, 170.0 (4xCO), 137.9, 137.7, 129.3, 129.2, 128.2, 127.9, 126.4, 126.1 (Ar), 69.6, 69.3, 66.7 (3xCH_2^{PEG}), 54.0, 53.9, 51.0 (3xαC), 41.1 (βC), 38.6 (CH_2^{PEG}), 37.4, 36.7 (2xβC), 35.7 (CH_2^{PEG}), 23.9 (γC), 22.7, 22.0 (2xδC). **MS (ESI):** m/z 585.2 $(M+H)^+$, $C_{31}H_{44}N_4O_7$ requires 584.3.

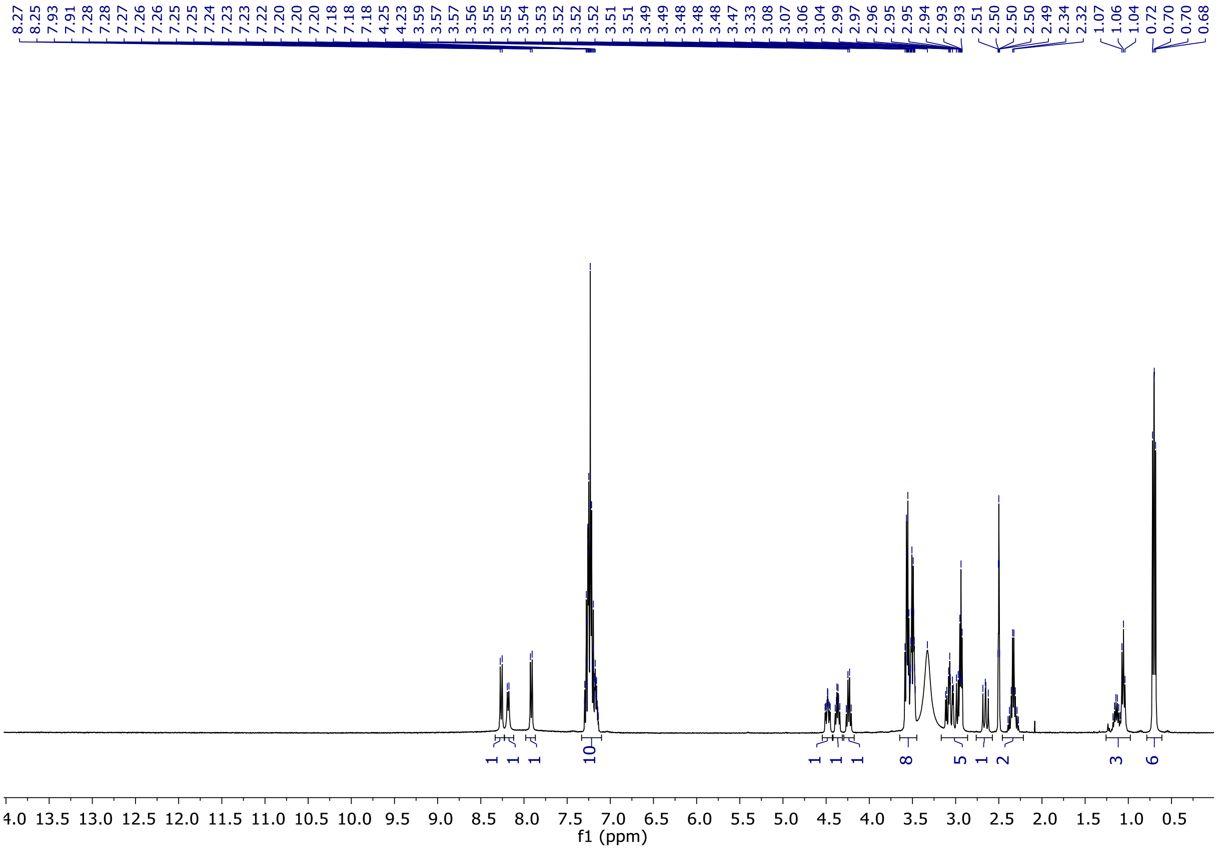

Figure S10. 400 MHz ^{1}H-NMR spectrum of amino-PEG-Lff in DMSO-d_6

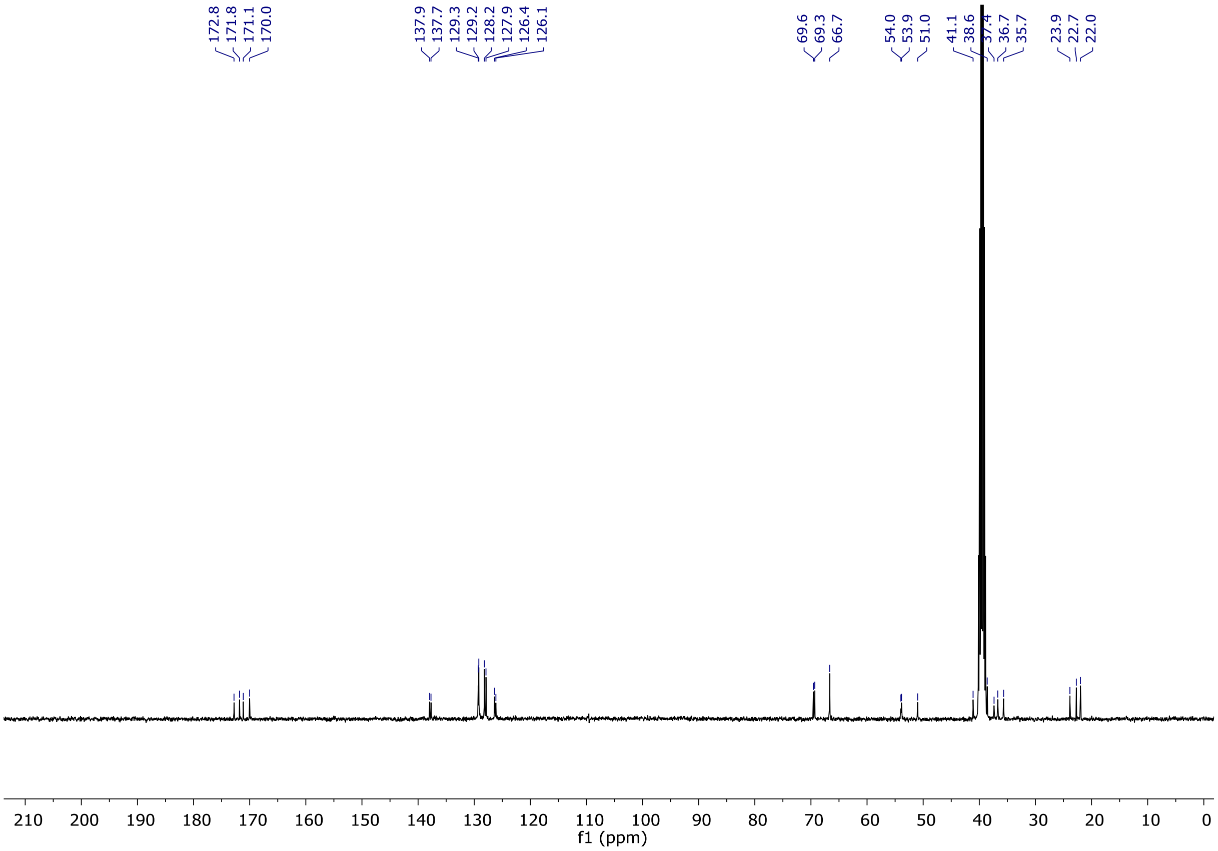

Figure S11. 400 MHz ^{13}C-NMR spectrum of amino-PEG-Lff in DMSO-d_6

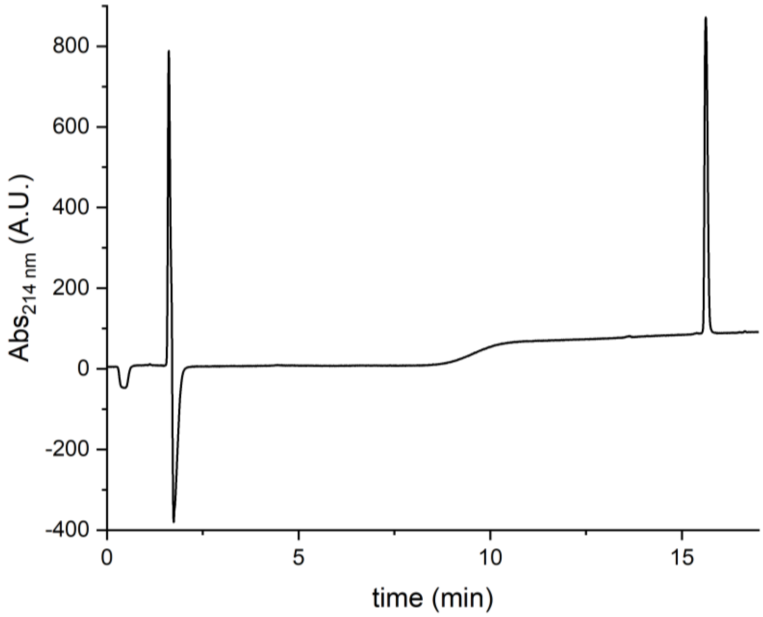

Figure S12. HPLC trace of purified amino-PEG-Lff.

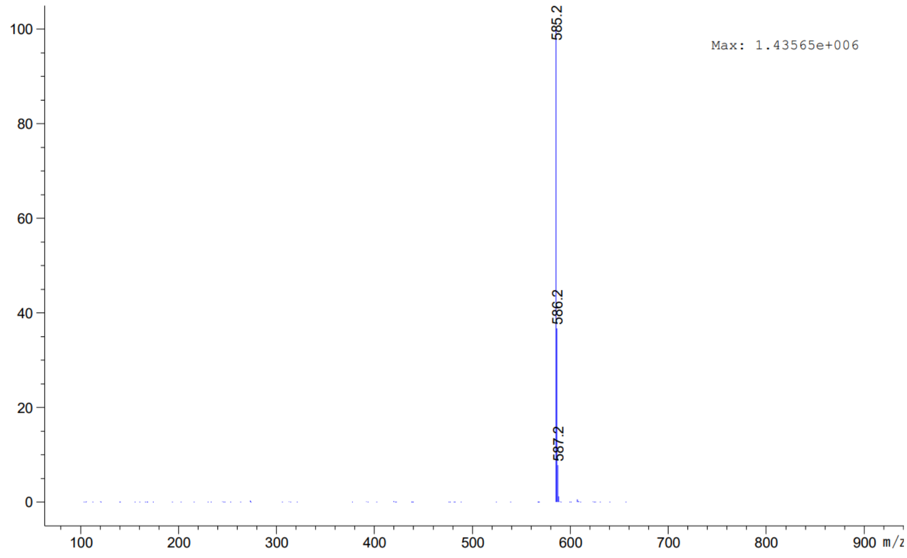

Figure S13. ESI-MS spectrum of amino-PEG-Lff in positive ion mode

4. Photographs of oxi-CNOs dispersed in the gel-precursor solution

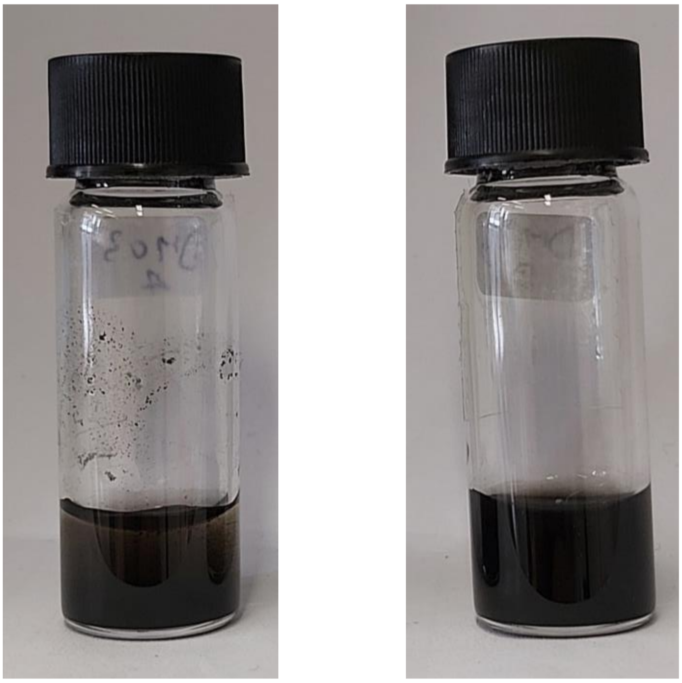

Figure S14. Photographs of oxi-CNOs (2.0 mg/ml) without (left) and with (right) the self-assembling tripeptide Lff in alkaline sodium phosphate solution after 20 minutes since ultrasonication (15 min).

5. TEM image of oxi-CNOs

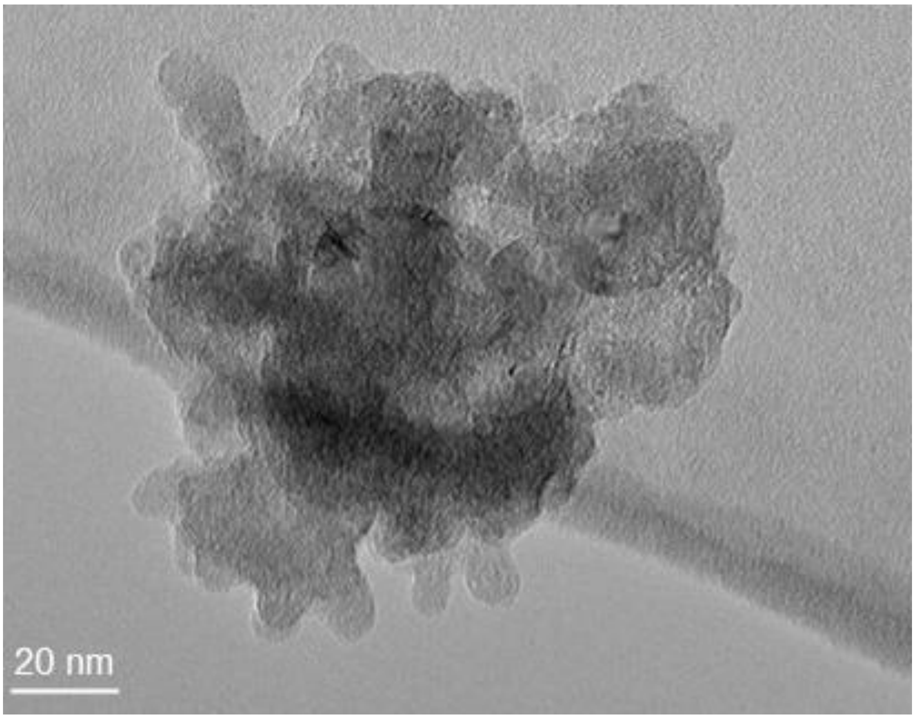

Figure S15. TEM micrograph of aggregated oxi-CNOs

6. Characterization of Lff-PEG-CNOs

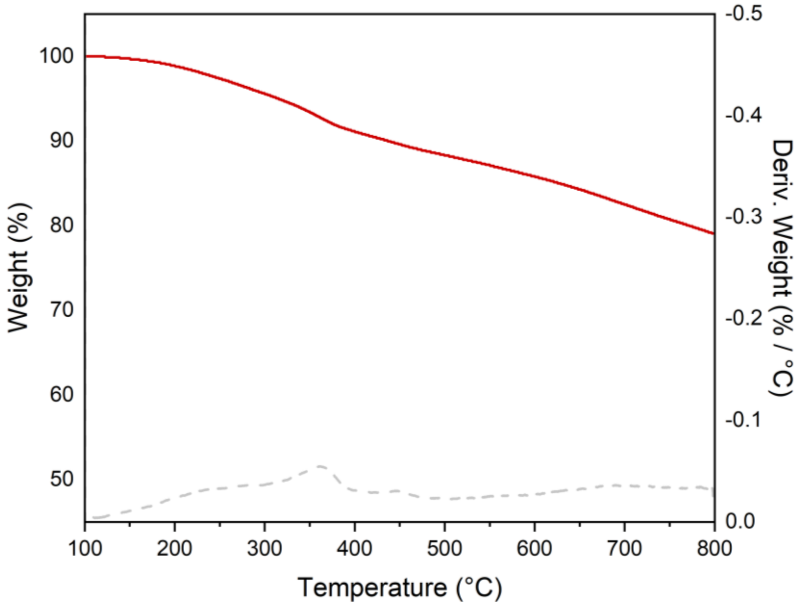

Figure S16. TGA data in a nitrogen atmosphere for Lff-PEG-CNOs

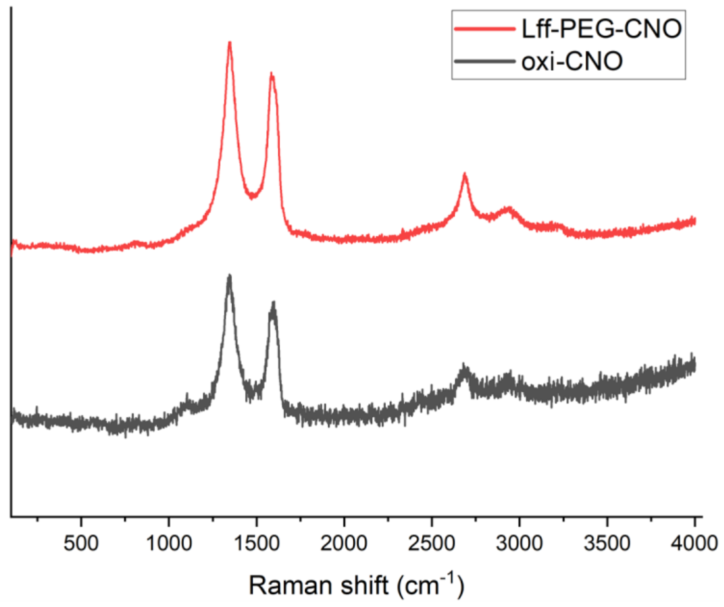

Figure S17. Raman spectra of Lff-PEG-CNOs and oxi-CNOs

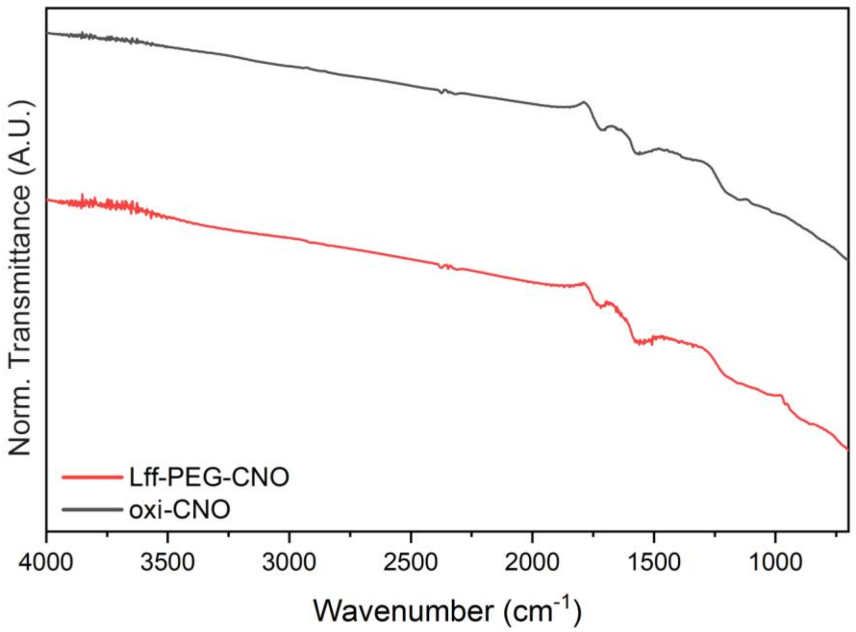

Figure S18. ATR-FTIR spectra of Lff-PEG-CNOs and oxi-CNOs

7. Photographs of Lff-PEG-CNOs dispersions in the gel-precursor solution

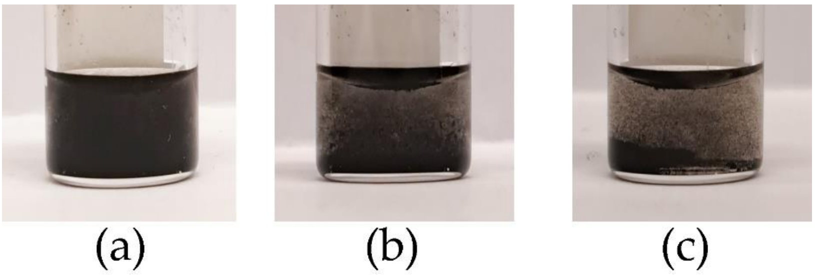

Figure S19. Photographs of Lff-PEG-CNOs dispersions in alkaline sodium phosphate buffer (2.0 mg/ml) after **(a)** 20 minutes, **(b)** 30 minutes, **(c)** 40 minutes since ultrasonication (15 min).

8. TEM images of covalent gel with Lff-PEG-CNOs

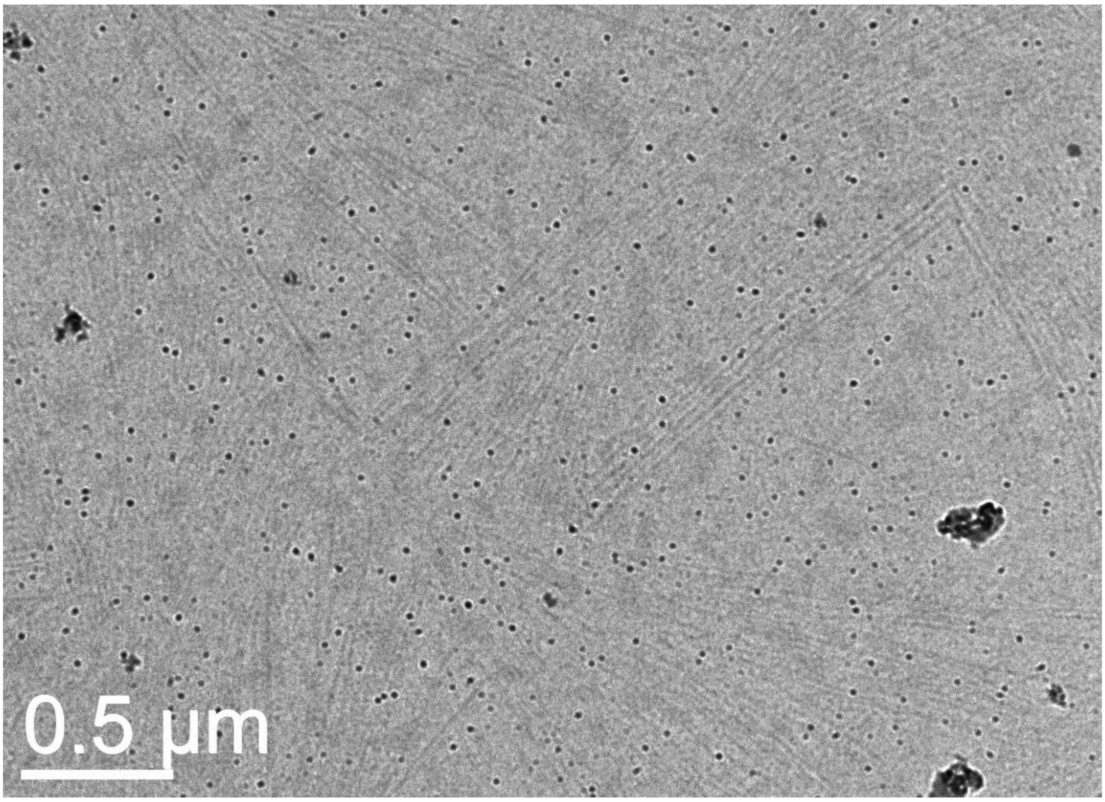

Figure S20. TEM image of Lff-PEG-CNOs covalent gel with Lff

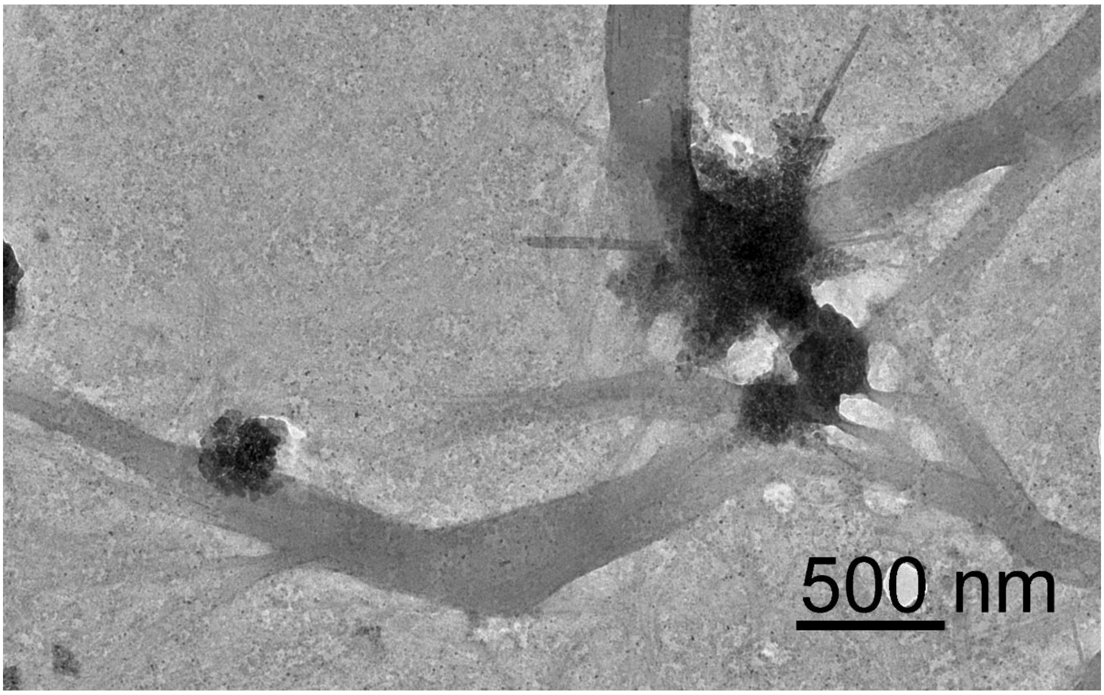

Figure S21. TEM image of Lff-PEG-CNOs covalent gel with Lff

9. Photographs of Lff-PEG-CNOs dispersions in the gel-precursor solution with the Lff tripeptide.

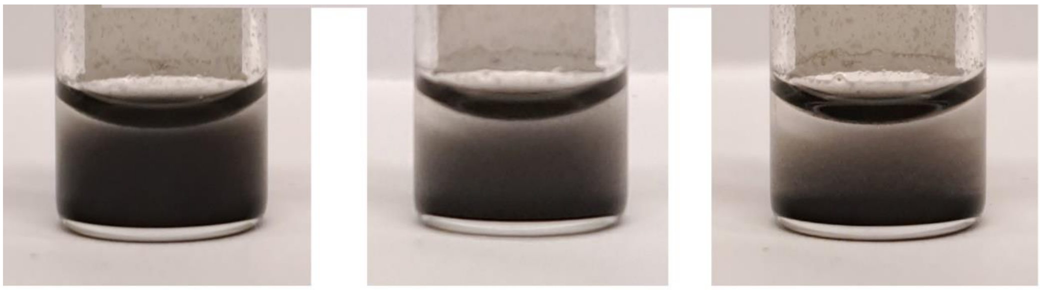

(a) (b) (c)

Figure S22. Photographs of Lff-PEG-CNOs dispersions (2.0 mg/ml) in pH 11.8 alkaline sodium phosphate buffer with Lff tripeptide after **(a)** 10 minutes, **(b)** 20 minutes, **(c)** 30 minutes since ultrasonication (15 min).

10. Oxi-CNOs release study

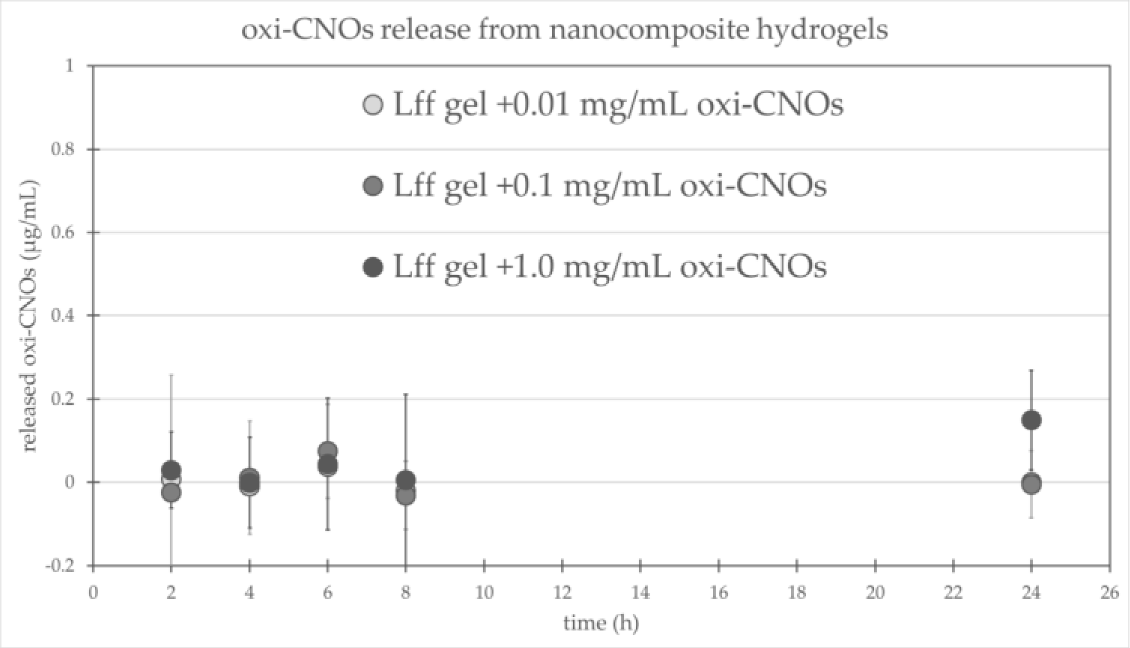

Figure S23. Oxi-CNOs release study form the nanocomposite hydrogels.
